# Exosomal miR-146a-3p from BEAS-2B cells alleviates NETs-Aggravated asthma by targeting HIF-1α to inhibit ferroptosis

**DOI:** 10.1016/j.redox.2026.104455

**Published:** 2026-10-26

**Authors:** Jianing Yang, Yunong Li, Zhien Li, Mingyue Liu, Yedong Xiang, Feifei Xu, Yulian Zhang, Guanhao Li, Jingmei Chai, Guanghai Yan, Chongyang Wang

**Affiliations:** a Jilin Key Laboratory for Immune and Targeting Research on Common Allergic, Diseases, Yanbian University, Yanji, 133002, PR China; b Department of Anatomy, Histology and Embryology, Yanbian University Medical, College, Yanji, 133002, PR China; c Department of Traditional Chinese Medicine, Yanbian University Medicine College, Yanji, 133002, PR China; d Yanbian University Agriculture College, Yanji, 133002, PR China

**Keywords:** Neutrophil extracellular traps, miR-146a-3p, Asthma, Ferroptosis, cGAS-sting

## Abstract

The molecular mechanism by which hypoxia-inducible factor 1-alpha (HIF-1α) regulates asthma pathogenesis through modulation of neutrophil extracellular trap (NET) formation remains poorly defined. Given that NETs act as core drivers of allergic asthma pathology by amplifying airway inflammation and inducing airway epithelial ferroptosis, this study aimed to elucidate the regulatory axis involving airway epithelial cell-derived exosomal miR-146a-3p. We proposed that exosomal miR-146a-3p directly targets HIF-1α to suppress the transcriptional activity of its downstream target gene *ELANE*, thereby blocking NETs formation and ultimately alleviating asthmatic airway inflammation. Using public transcriptomic data from asthma patients, we identified miR-146a-3p as significantly downregulated in asthmatic tissues. BEAS-2B cell-derived exosomes were isolated via differential centrifugation. Employing a PMA-induced neutrophil inflammation model and an HDM/NETs-challenged asthmatic mouse model, we systematically demonstrated that BEAS-2B cell-derived exosomes function as efficient delivery vehicles for miR-146a-3p, transporting it into neutrophils. By targeting and inhibiting HIF-1α nucleocytoplasmic translocation and its transcriptional activation capacity, exosomal miR-146a-3p significantly downregulated the expression of NE—key mediator of NET formation—thereby blocking aberrant NETs release. Moreover, we confirmed that NETs reduction modulates the cGAS-STING signaling pathway, reverses the aberrant expression patterns of the ferroptosis regulatory proteins GPX4, SLC7A11, and ACSL4, maintains cellular iron homeostasis, and subsequently alleviates airway epithelial ferroptosis, ultimately ameliorating asthmatic airway inflammation.

Collectively, this study reveals a novel mechanism by which excessive NET accumulation exacerbates asthma pathology through cGAS-STING-mediated airway epithelial ferroptosis. Furthermore, it identifies BEAS-2B cell-derived exosomal miR-146a-3p as a key regulatory molecule and potential therapeutic target, offering a new theoretical framework and intervention strategy for understanding and treating allergic asthma.

## Highlights

• BEAS-2B-derived exosomal miR-146a-3p transfers to neutrophils.
• Exosomal miR-146a-3p targets HIF-1α, suppresses ELANE and limits NET release.
• Reduced NETs inhibit cGAS–STING signaling and ferroptosis.
• Exosomal miR-146a-3p alleviates airway inflammation in NETs-exacerbated asthma.

## Graphical abstract

**Figure:**
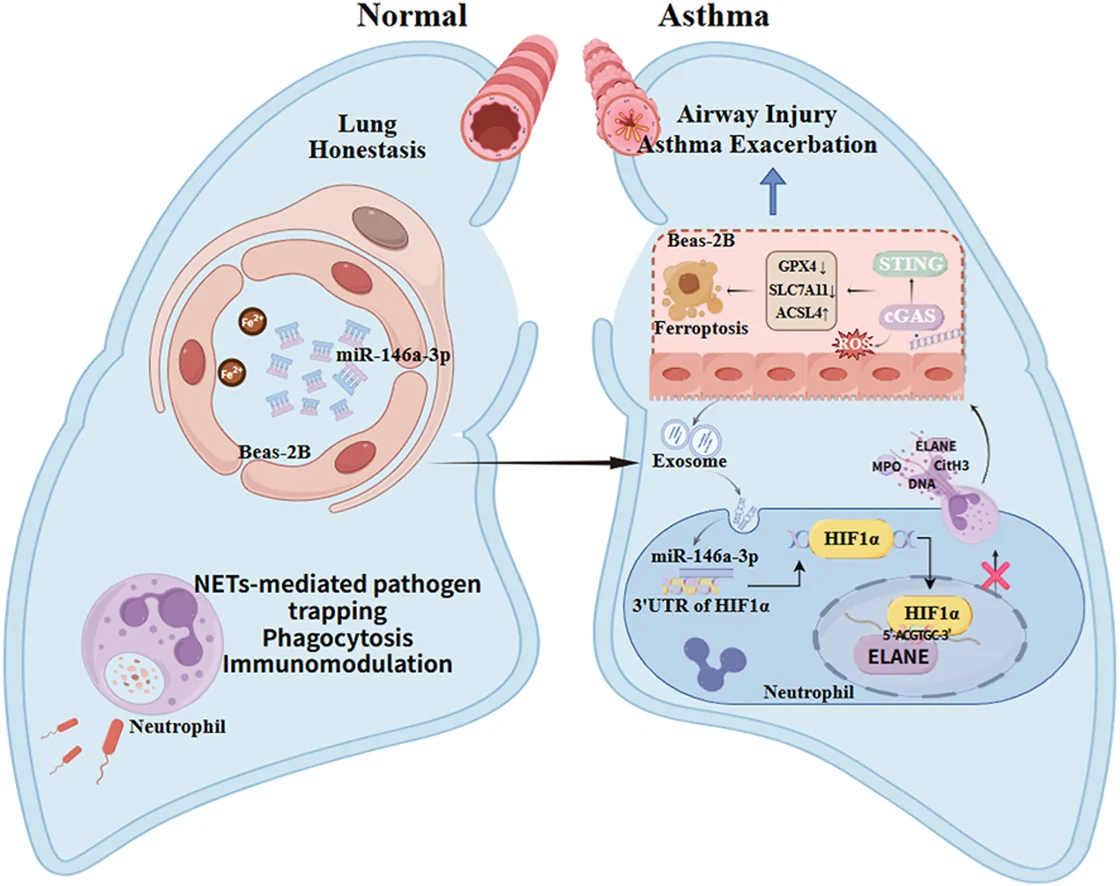

## 1 Introduction

Asthma is a common chronic inflammatory airway disease, whose complex pathological mechanisms have not been fully elucidated. Although traditional perspectives focus on Th2 immune responses and eosinophil infiltration [1], [2], the role of neutrophils and neutrophil-mediated immune responses in subtypes such as severe asthma and corticosteroid-resistant asthma is increasingly recognized [3]. Among these, the formation of NETs is a key effector mechanism of neutrophils. NETs are composed of extracellular DNA (eDNA), citrullinated histone H3 (Cit-H3), and granular proteins such as myeloperoxidase (MPO) and neutrophil elastase (NE) [4]. In a pro-inflammatory feedback loop, NET-derived damage-associated molecular patterns promote neutrophilic chemotaxis by releasing CXCL1/2/8 chemokines in a TLR4/NF-κB-dependent manner [5]. Furthermore, NETs worsen asthma pathophysiology by activating inflammatory cascades in airway epithelial cells and eosinophils as well as directly disrupting the epithelial barrier [6]. They are a critical factor driving airway hyperresponsiveness and tissue remodeling in asthma [7]. Notably, HIF-1α, a core regulator of cellular adaptive responses to hypoxia and inflammation, has been increasingly recognized as a pivotal mediator in asthma pathogenesis [8]. Accumulating evidence indicates that HIF-1α is aberrantly upregulated in the airways of asthma patients and murine asthma models [9]. Its overexpression promotes neutrophil recruitment, activation, and the formation of NETs [10], a process closely linked to airway inflammation exacerbation and tissue damage. Studies have also found that targeting HIF-1α can effectively mitigate NETs formation [11], highlighting its potential as a therapeutic target for disease.

In recent years, the role of a novel mode of cell death termed ferroptosis in the pathogenesis of asthma has begun to be revealed [12]. Ferroptosis is an iron-dependent form of regulated cell death characterized by the abnormal accumulation of lipid peroxides [13]. Han and Zeng [14], [15] demonstrated that IL-6 and HDM can trigger ferroptosis in airway epithelial cells, thereby promoting the production of lipid peroxides and inflammatory mediators. Emerging evidence has subsequently indicated that in non-type 2 asthma, Th1/Th17 polarization promotes ferroptosis via the IL-17/NF-κB pathway, and the crosstalk between ferroptosis and autophagy amplifies neutrophilic inflammation [16]. However, the upstream signals driving ferroptosis in asthma remain unclear. Emerging evidence suggests that NETs are not merely inflammatory mediators but may also, by activating cGAS–STING pathway and regulating downstream oxidative stress and lipid metabolism [17], induce ferroptosis in airway epithelial cells. This forms a detrimental axis of NETs–cGAS–STING–ferroptosis, exacerbating the progression of asthma. Therefore, identifying intervention strategies capable of targeting this pathological axis is of significant importance.

Exosomes derived from normal BEAS-2B, owing to their inherent biocompatibility and targeting specificity, serve as ideal vehicles for delivering therapeutic molecules [18]. miRNAs are key functional components of exosomes. Beyond their role as biomarkers for early disease diagnosis [19], miRNAs regulate post-transcriptional gene expression through binding to the 3′ untranslated region (UTR) of target mRNAs [20]. Sara et al. [21] discovered that serum exosomal miRNAs may serve as surrogate biomarkers for asthma phenotypes and severity. Fang et al. [22] observed that mesenchymal stromal cell-derived exosomes could inhibit Th2 cell differentiation via miR-146a-5p and prevent type 2 innate lymphoid cell-driven allergic airway inflammation. Studies have found that miR-146a-3p can effectively suppress the NF-κB pathway by targeting Nron, thereby mitigating macrophage inflammation [23]. We found that miR-146a-3p is significantly downregulated in asthma, exhibiting a strong negative correlation with airway neutrophil infiltration, which indicates a potential protective role against neutrophilic inflammation in this context. However, the regulatory mechanism of miR-146a-3p on NETs remains unclear, and whether this regulatory effect is associated with the HIF-1α-mediated pathway has not been elucidated. We hypothesize that miR-146a-3p, enriched in BEAS-2B-derived exosomes, may protect airway epithelial cells by directly or indirectly inhibiting neutrophil activation and NETs formation, thereby reducing the triggering factors at their source and blocking the NETs-induced ferroptosis process.

This study aims to investigate whether exosomes derived from BEAS-2B cells can deliver miR-146a-3p to inhibit neutrophil NETs formation, block NETs-mediated activation of the cGAS-STING pathway, thereby attenuating ferroptosis in airway epithelial cells, and ultimately alleviate airway inflammation and tissue damage in asthma.

## 2 Materials and methods

### 2.1 Pathway and cell death mode analysis

Asthma-associated targets were retrieved from the GeneCards (https://www.genecards.org/), OMIM (https://www.omim.org/), and TTD (http://db.idrblab.net/ttd/) databases using the keyword "asthma". After removing duplicates, a total of 9, 036 targets were identified. These targets were then uploaded to the STRING database (v12.0) for protein–protein interaction (PPI) analysis, with the species limited to *Homo sapiens* and a minimum interaction score set at ≥ 0.4. The resulting PPI network was imported into Cytoscape (v3.10.2), and topological analysis was conducted using the cytoHubba plugin. Core targets were defined by integrating degree, betweenness centrality, and closeness centrality. GO enrichment analysis, covering biological process (BP), cellular component (CC), and molecular function (MF), alongside KEGG pathway analysis, was performed via Metascape (https://metascape.org/), with a significance threshold of adjusted *P* < 0.05. Core targets were intersected with ferroptosis-, cuproptosis-, and necroptosis-related genes retrieved from the same databases to identify overlapping candidates engaged in multiple cell death modalities.

### 2.2 NETs regulatory mechanism analysis

Targets associated with asthma, ferroptosis, and NETs were obtained from GeneCards, OMIM, and TTD. The ferroptosis–NET intersection yielded 1193 common targets, which were analyzed via Metascape for KEGG pathway enrichment. Next, the three-way intersection of asthma-, ferroptosis-, and NETs-related targets was performed, identifying 810 shared targets. These targets were also analyzed by Metascape for KEGG pathway enrichment to decipher the potential regulatory networks of NETosis in asthma.

### 2.3 Differential cell counting

BALF samples were prepared as smears on glass slides. After air-drying, the smears were fixed with methanol for 20 s. Subsequently, the slides were sequentially stained with Solution I from a Diff-Quik Rapid Staining Kit (G1541, Solarbio, Beijing, China) for 5–10 s, followed by Solution II for 10–20 s. Following a rinse with distilled water, the smears were mounted with resin-based mounting medium. Digital image acquisition and analysis were performed using the SQS-40P stage scanning system.

### 2.4 Animal experiments

The 6-8-week-old male C57BL/6J mice were purchased from the Animal Center of Yanbian University. All mice were housed in specific pathogen-free conditions. The room temperature is 22 ± 2 °C, the relative humidity is 50%-60%, and the light and dark cycle is 12 h. Maintain a certain temperature and humidity to ensure sufficient food and moisture. All animals were used in accordance with the Laboratory Animal Use License (SYXK (JI) 2020-0009) of the Jilin Provincial Department of Science and Technology. All animal experimental procedures have been approved by the Animal Care and Utilization Committee of Yanbian University (approval number: YDSY20260814001).

### 2.5 Animal treatments

The mice were intranasally sensitized with 50 μg of HDM (XPB46D3A4, Greer Laboratories, Lenoir, NC, USA) on day 0, followed by daily intranasal HDM challenge (50 μg per mouse) from day 7 to day 13 to induce airway hyperresponsiveness and recapitulate the asthmatic phenotype. To determine the appropriate dosage for exogenous NETs intratracheal instillation, preliminary experiments were performed: mice were sacrificed 12 h after HDM challenge under pentobarbital sodium anesthesia, and serum as well as BALF were harvested. The MPO-DNA levels in serum and BALF were quantified using an ELISA kit, and these values were used to calculate the working dose of exogenous NETs for in-vivo administration.

Mice were randomly assigned to four groups: control group, HDM group, HDM + NETs group, HDM + NETs + BAY 87-2243 group and HDM + NETs + sEV-miR146a-3p. To maintain the same intervention route as sEV administration, BAY 87-2243 (a selective HIF-1α inhibitor; Sigma, Cat. No. SML2384) was administered via tail-vein injection at 1 mg per mouse once daily from day 7 to day 13. On the final day of model establishment (day 14), following pentobarbital sodium anesthesia, mice in the control group received intratracheal instillation of an equal volume of normal saline. Mice in the HDM + NETs group, HDM + NETs + BAY 87-2243 group and HDM + NETs + sEV-miR146a-3p were subjected to intratracheal instillation of exogenous NETs (50 μL NET suspension containing 2 μg NET-DNA) isolated from mouse bone marrow-derived neutrophils. All animals were sacrificed 24 h after exogenous NETs instillation. Lung tissues, serum and BALF were collected for subsequent experimental analyses.

### 2.6 Isolation and collection of NETs

After euthanizing the mice, the femurs and tibiae were aseptically isolated. The bone marrow cavity was flushed with pre-cooled, sterile PBS to collect the cell suspension. Neutrophils were isolated using Ly6G-coated magnetic beads, followed by treatment with red blood cell lysis buffer to remove residual erythrocytes. Neutrophils were resuspended in RPMI 1640 medium (Biological Industries, #01-101-1A) supplemented with 10% FBS (SbjBio, BC-SE-FBS07) and seeded into 6-well plates at a density of 1 × 10^6^ cells/well. After incubation at 37 °C and 5% CO_2_ for 1 h, PMA was added to a final concentration of 100 nM, and the cells were incubated for a further 4 h to induce NET formation.

After PMA stimulation, the culture medium was gently aspirated and discarded, and the cells were washed twice with pre-cooled PBS to remove non-adherent material. An appropriate volume of pre-cooled PBS was added, and NETs were gently scraped from the bottom of each well using a cell scraper and collected into 15 mL centrifuge tubes. The suspension was centrifuged at 450 × g for 10 min at 4 °C to remove intact cells and debris. The supernatant was carefully transferred to 1.5 mL microcentrifuge tubes and centrifuged again at 18,000 × g for 10 min at 4 °C to pellet the DNA. The supernatant was discarded, and the remaining pellet was resuspended in ice-cold PBS. The concentration of free DNA was quantified using the Quant-iT PicoGreen dsDNA Assay Kit (Thermo Fisher ScientificM, P7589) according to the manufacturer's instructions, which served as a quantitative indicator of NETs. The NET samples were either used immediately or stored at −80 °C for preservation.

HL-60 cells were purchased from ATCC (10801 University Avenue, Manassas, VA 20110, United States) and routinely maintained in HL-60 cell-specific culture medium (iCell-h098-001b) supplemented with 20% FBS. For differentiation, cells were seeded into fresh medium at a density of approximately 2 × 10^5^ cells/mL, and DMSO was added as a differentiation inducer to a final concentration of 1.25%. The cells were then cultured for 4–5 days at 37 °C under 5% CO_2_. During this period, the cells were counted every other day, and the medium was replaced with fresh medium containing the same concentration of DMSO to maintain the cell density below 1 × 10^6^ cells/mL. Observation under an inverted microscope confirmed successful differentiation, characterized by reduced cell volume, a decreased nuclear-to-cytoplasmic ratio, and lobulated (rod-shaped or segmented) nuclei. The differentiated HL-60 cells were washed and resuspended in serum-free RPMI 1640 medium, and PMA was added to a final concentration of 100 nM, followed by stimulation for 3–4 h to induce NET formation.

### 2.7 Histological analysis

After lung tissue extraction, the lung tissues were fixed in 4% paraformaldehyde for 7 days. Following overnight rinsing under running water, the tissues were embedded in paraffin and sectioned into 4-μm-thick slices. Hematoxylin and eosin (H&E; G1120, Solarbio, Beijing, China) staining was performed to observe inflammatory cell infiltration in the lung tissues. Periodic acid-Schiff (PAS; G1281, Solarbio, Beijing, China) staining was used to evaluate goblet cell hyperplasia and mucus secretion. Masson's trichrome staining (G1340, Solarbio, Beijing, China) was applied to distinguish collagen fibers from elastic fibers. Finally, the lung tissue sections were analyzed using a biopsy scanning system (SQS-40P, teksqray, Shenzhen, China).

To eliminate subjective bias, two independent researchers performed the scoring. A semiquantitative histological lung inflammation scoring system ranging from 0 to 5 points was adopted. The scoring items included the inflammatory area, inflammatory cell types, neutrophil infiltration in alveolar cavities, hemorrhage, and thickening of alveolar septa. The scoring criteria are presented in Table 1. The final score could be assigned by a blinded observer using representative H&E-stained lung sections, with higher scores indicating more severe lung inflammation.

**Table 1:** Histopathological evaluation of pulmonary inflammation. Representative hematoxylin and eosin (H&E)-stained lung sections from each experimental group were examined under light microscopy to evaluate the severity of inflammatory injury. Inflammation was scored semi-quantitatively on a 0–5 scale, taking into account six histopathological features.

Score	Degree of inflammation	Inflammatory area/distribution	Inflammatory cell types and infiltration	Neutrophil infiltration in alveolar cavities	Hemorrhage	Thickening of alveolar septa	Other/architectural changes
0	No visible pathological changes	No inflammation	No inflammatory cell infiltration	None	None	None/normal	Intact alveolar structure
1	Minimal inflammation	Rare inflammatory cells scattered in the lunginterstitium	Rare scattered inflammatory cells	None	None	Normal or only minimally thickened	_
2	Mild inflammation	Small foci of inflammatory cell infiltration inthe interstitium, occasionally extending intoalveolar spaces	Small focal inflammatory cell infiltration	Not specified	None or only minimal focal hemorrhage	Mildly thickened	_
3	Moderate inflammation	More frequent or confluent interstitialinflammatory infiltration, occasionallyinvolving alveolar spaces	More frequent/confluent interstitialinflammatory infiltration	A small number	Focal hemorrhage	Moderately thickened	_
4	Marked inflammation	Extensive interstitial and intra-alveolarinflammatory cell infiltration	Extensive inflammatory cell infiltration	Moderate	Multifocal hemorrhage	Markedly thickened	_
5	Severe inflammation	Diffuse inflammatory infiltration and consolidation	Diffuse inflammatory infiltration	Abundant	Extensive hemorrhage	Severely thickened	Architectural distortion

### 2.8 Immunohistochemistry

Immunohistochemical staining for NE was performed on lung tissue sections. After overnight incubation at 4 °C with primary antibodies (rabbit anti-Neutrophil Elastase), sections were treated with secondary antibodies using a PV9000 immunohistochemistry kit (Zhongshan Jinqiao). Stained sections were digitally analyzed with a SQS-40P slide scanning system (TEKSQRAY).

### 2.9 LDH release assay

BEAS-2B cells were seeded at 3 × 10^4^ cells/well in 96-well plates. Cell-free supernatant was collected after corresponding treatments. Maximum LDH release control (cells treated with lysis buffer) and untreated negative control were set in parallel, incubated according to LDH Cytotoxicity Assay Kit (C0016, Beyotime, Shanghai, China) instructions at room temperature, absorbance read at 490 nm.

### 2.10 Detection of lipid peroxidation-related indicators

Lipid peroxidation is a hallmark feature of ferroptosis. To validate changes in related markers, the level of malondialdehyde (MDA) was assessed using the MDA Assay Kit (S0131S, Beyotime, Shanghai, China) according to the manufacturer's instructions; the levels of oxidized glutathione (GSSG) and total glutathione (T-GSH) were measured with a Total GSH/GSSG Colorimetric Assay Kit (S0053S, Beyotime, Shanghai, China); the level of 4-HNE (4-hydroxynonenal) was detected using a 4-HNE ELISA Kit (E-EL-0128, Elabscience, Wuhan, China).

### 2.11 CCK-8

BEAS-2B cells in logarithmic growth phase were selected and seeded in 96-well plates at 3 × 10^4^/ml, 100μL/well. Each set is set up with 4 compound wells and the edges are filled with sterile PBS. Place in a 37 °C 5% CO_2_ incubator and incubate for 6 h. Subsequently, the medium was added to the medium at 4 μL/well according to the concentration gradient and incubated in a 37 °C 5% CO_2_ incubator for 24 h. Next, 10 μL of CCK-8 (K1018, APExBIO) to each well and incubated for 2 h in a 37 °C 5% CO_2_ incubator protected from light. Blank wells containing medium only and untreated control groups were included. The absorbance at 450 nm was detected using a microplate reader. Finally, cell viability was calculated according to the formula (OD value of drug group/OD value of control group) × 100%.

### 2.12 Immunofluorescence

Lung tissue sections were deparaffinized in xylene and a graded alcohol series. The sections were placed in 0.01 M citrate buffer (pH 6.0), which was heated and boiled above 95 °C in an autoclave. After allowing the solution to cool naturally to below 30 °C, the sections were incubated with 0.5% Triton X-100 for 30 min. Subsequently, blocking was performed with 5% bovine serum albumin (BSA) for 1 h. The lung tissue sections were incubated overnight at 4 °C with the following primary antibodies: anti-SP-C (ab90716, Abcam), ACSL4 (Cat. # MA5-31548, ThermoFisher, MA, USA), and ZO-1 (ab307799, Abcam). The next day, AlexaFluor 488-conjugated goat anti-rabbit IgG (Cat. # A11001, Thermo Fisher Scientific, MA, USA) and Cy3®-conjugated goat anti-mouse IgG H&L (ab97035, Abcam) were used as secondary antibodies and incubated at room temperature for 2 h. After three washes with PBS buffer, nuclei were counterstained for 2 h with an anti-fade mounting medium containing DAPI (P0131, Beyotime, Shanghai, China). Fluorescence imaging was performed using a Cytation 5 fluorescence microscope (BioTek, VT, USA).

After fixation with 4% paraformaldehyde (PFA, Solarbio, P1110, Beijing, China), samples were permeabilized with 0.2% Triton X-100 at room temperature for 30 min. Blocking was performed using 5% BSA for 1 h. Subsequently, the samples were incubated overnight at 4 °C with the following primary antibodies: GPX4 (DF8701, Affinity), cGAS (A8335, Abclonal), STING (AB3499, Abways), IRF3 (A2172, Abclonal), and HIF-1α (ab179483, Abcam). The next day, corresponding secondary antibodies-Alexa Fluor 488-conjugated goat anti-rabbit IgG (Cat. # A11001, Thermo Fisher Scientific, USA) and Cy3®-conjugated goat anti-mouse IgG H&L (ab97035, Abcam) — were applied and incubated at room temperature for 2 h. Finally, after three 5-min washes with PBS, cell nuclei were counterstained with anti-fade mounting medium containing DAPI for 2 h. Fluorescence imaging was performed using a Cytation5 microscope.

### 2.13 Western blot

Western blot was used to measure protein expression levels. After extracting the cells and lung tissue proteins, they were lysed using RIPA lysate. The concentration of the proposed protein was detected by bicinchoninic acid (P0010S, Beyotime) kit. Next, 20 μg of protein was separated using SDS-PAGE and transferred to a PVDF membrane (Millipore, Billerica, MA, USA). We used 5% skim milk powder for closure. Subsequently, the primary antibody was incubated at 4 °C overnight and the secondary antibody was incubated for 1h at room temperature the next day. The primary antibodies we needed include anti-GPX4 (DF8701, Affinity, 1:1000), anti-ACSL4 (Cat. # MA5-31548, ThermoFisher, 1:1000), anti-SLC7A11 (BY0161, Abways, 1:1000), anti-cGAS (A8335, Abclonal, 1:1000), anti-STING (AB3499, Abways, 1:1000), anti-IRF3 (A2172, Abclonal, 1:500), anti-p-IRF3 (AP0995, Abclonal, 1:1000), anti-TBK1(A3458, abclonal, 1:1000), anti-p-TBK1(AP1026, abclonal, 1:1000), HIF-1α (ab179483, Abcam), anti-β-tubulin (A12289, Abclonal, 1:5000), anti-citrullinated histone H3 (Cit-H3, ab5103, Abcam, 1:1000), anti-PAD4 (ab96758, Abcam, 1:1000), anti-CD9 (ab307085, Abcam, 1:1000), anti-TSG101 (ab125011, Abcam, 1:1000), anti-CD63 (ab271286, Abcam, 1:1000), anti-CD81 (ab219209, Abcam, 1:1000), anti-β-actin (#3700, Cell Signaling Technology, 1:1000). The secondary antibodies used in the experiment were:anti-rabbit IgG (H + L) (DyLight™ 800 4X PEG Conjugate) (#5151, CST, 1:5000) and anti-mouse IgG (H + L) (DyLight™ 800 4X PEG Conjugate) (#5257, CST, 1:5000).

### 2.14 FerroOrange staining

Intracellular labile iron (Fe^2+^) levels were measured using the fluorescent probe FerroOrange. BEAS-2B cells were seeded into appropriate culture plates or dishes and grown to approximately 70–80% confluence before the indicated treatments. After treatment, the culture medium was removed, and cells were washed once with PBS to remove residual medium and serum. FerroOrange working solution was prepared immediately before use by diluting the stock solution in HBSS buffer (without calcium and magnesium) to a final concentration of 1 μmol/L. Cells were then incubated with the FerroOrange working solution at 37 °C for 1 h in the dark. After incubation, the dye-containing solution was removed, and cells were washed two to three times with HBSS buffer or serum-free medium to remove unincorporated probe. Fluorescence images were captured using a Cytation 5 imaging system (BioTek, USA) with excitation/emission wavelengths of approximately 543/580 nm. Acquisition parameters, including exposure time, gain, and magnification, were kept constant across all groups to allow direct comparison. The MFI per field or per cell was quantified using the accompanying Gen5 software. All procedures were performed in the dark, and each experiment was repeated at least three times independently.

### 2.15 ROS detection

Intracellular reactive oxygen species (ROS) levels were measured using the cell-permeable fluorescent probe 2′,7′-dichlorodihydrofluorescein diacetate (DCFH-DA; 10 μM, S0033S, Beyotime, Shanghai, China). For imaging-based detection, cells were seeded into appropriate culture plates or dishes and cultured to the desired confluence. After the indicated treatments, the culture medium was removed, and cells were washed once with serum-free medium. DCFH-DA working solution was prepared immediately before use by diluting the stock solution in serum-free medium to a final concentration of 10 μM. Cells were then incubated with the DCFH-DA working solution at 37 °C for 20 min in the dark. After incubation, the dye-containing solution was removed, and cells were washed three times with serum-free medium to remove unincorporated probe. Fluorescence images were captured using a Cytation 5 imaging system (BioTek, USA) with excitation/emission wavelengths of approximately 488/525 nm. Acquisition parameters, including exposure time and gain, were kept constant across all groups to allow direct comparison. Mean fluorescence intensity (MFI) per field or per cell was quantified using the accompanying Gen5 software.

For quantitative analysis of total ROS by flow cytometry, BEAS-2B cells were seeded into culture plates and grown to approximately 70–80% confluence before the indicated treatments. After the indicated treatments, cells were gently harvested with a cell scraper and collected directly into DCFH-DA working solution (10 μM, prepared in serum-free medium). The cell suspension was then incubated at 37 °C in the dark for 20 min, with gentle shaking every 5 min to ensure that the cells were evenly and fully stained. After incubation, the cells were washed twice with serum-free medium or PBS to remove unincorporated probe, and resuspended in serum-free medium at an appropriate density (typically 1 × 10^6^ cells/mL). Single-cell suspensions were analyzed on a CytoFlex flow cytometer (Beckman Coulter, USA) using the FITC channel (excitation 488 nm, emission 525 nm). Data were analyzed using CytExpert software, and ROS levels were expressed as the MFI of DCF fluorescence or as the percentage of DCF-positive cells. All experiments were performed in the dark and repeated at least three times independently.

### 2.16 Transfection

Plasmids for HIF-1α, ELANE and TSG101 overexpression or shRNA targeting were purchased from RIBOBIO (Guangzhou, China). dHL-60 cells were transfected using Lipofectamine 3000 (Invitrogen, Carlsbad, CA, USA) according to the manufacturer's instructions. Cells were harvested 24 h post-transfection for RNA/protein analysis.

### 2.17 RT-qPCR

To detect changes in the expression of *HIF1A* and NE at the nucleic acid level, we extracted total RNA using Fastking (Tiangen, DP451, Beijing, China). Complementary DNA (cDNA) was synthesized using the FastKing RT kit (Tiangen, KR118-02). Real-time quantitative polymerase chain reaction (RT-qPCR) was performed on an Azure Cielo 6 system (Azure, Dublin, CA, USA) using the SYBR Green RT-qPCR Kit (Tiangen, KR123). The PCR program is initially denatured at 95 °C for 15 min, followed by 40 cycles at 94 °C for 10 s, 55 °C for 20 s, and 72 °C for 20 s. The primers were obtained from Sangon Biotech (Shanghai, China), with their details provided in Table 2.

**Table 2:** 

Name	Sequence
Sting forward	5’-GCCCTGTCACTTTTGGTCCT-3’
Sting reverse	5’-TGGAGTATGGCATCAGCAGC-3’
IRF3 forward	5’-CAATTCCTCCCCTGGCTAGA-3’
IRF3 reverse	5’-CTGAACCTCGTTCGGCTCTC-3’
Tbk1forward	5’-GTACGGTGGCTGGTTGAACT-3’
Tbk1 reverse	5’-CTTGTGGACGGTCTCGTTGT-3’
HIF1A forward	5’-CCCAATGTCGGAGTTTGGAAAA-3’
HIF1A reverse	5’-TGTGCAGTGCAATACCTTCCA-3’
ELANE forward	5’-CGTGGCGAATGTAAACGTCC-3’
ELANE reverse	5’-ATTAGCCCGTTGCAGACCAA-3’
VEGFA forward	5’-CTCCACCATGCCAAGTGGTC-3’
VEGFA reverse	5’-CGCTTTCGTTTTTGCCCCTT-3’
GLUT1 forward	5’-TCTCGAAACTGGGCAAGTCC-3’
GLUT1 reverse	5’-TCACCCACATACATGGGCAC-3’
PGK1 forward	5’-CGGGGAACCAAAGCTCTCAT-3’
PGK1 reverse	5’-ATTGCTGAGAGCATCCACCC-3’

### 2.18 Downstream transcription factor screening of miR-146a-3p

Target genes of miR-146a-3p were predicted using TargetScan (https://www.targetscan.org/) and miRDB (https://mirdb.org/), with the species limited to *Homo sapiens*. The intersection of both prediction results yielded 393 consensus targets, which were subjected to GO and KEGG enrichment analyses in Metascape. To identify transcription factors (TFs) among these targets, the 393 genes were overlapped with the TF-target databases TFTarget and ENCODE. Furthermore, the binding potential of miR-146a-3p to the candidate TFs *RB1* and *HIF1A* was assessed using miRDB, including target score analysis, binding site visualization, and cross-species seed region conservation analysis.

### 2.19 Transcription Factor–Promoter binding site analysis

The expression correlation between *HIF1A* and *ELANE*, and *MPO* in lung tissue were analyzed via the GEPIA database (http://gepia.cancer-pku.cn/) with the organ filter set to "Lung". To investigate transcriptional regulation, the 2000 bp promoter region upstream of the transcription start site (TSS) of human ELANE was retrieved from NCBI Gene. The sequence was submitted to the JASPAR database (https://jaspar.genereg.net/) to predict potential binding sites of the transcription factor *HIF1A* within the *ELANE* promoter.

### 2.20 Protein–protein docking of HIF-1α and NE

The three-dimensional structures of HIF-1α (PDB ID: 1H2K) and NE (PDB ID: 6SMA) were downloaded from the RCSB PDB database (https://www.rcsb.org/) in PDB format. Preprocessing was performed using PyMOL (v3.0.3), including the removal of water molecules and heteroatoms, as well as the addition of hydrogen atoms. Molecular docking between HIF-1α and NE was then conducted via the HADDOCK 2.4 server (https://wenmr.science.uu.nl/haddock2.4/) with default parameters. The resulting docking models were visualized and rendered using PyMOL 3.0.3.

### 2.21 sEV isolation and characterization

BEAS-2B cells were cultured in exosome-depleted serum medium (41210ES76, Yeasen Biotechnology, Shanghai, China) until reaching approximately 80% confluence. The conditioned medium was collected into centrifuge tubes. The conditioned medium was first centrifuged at 4 °C, 2000 × g for 30 min. The supernatant was then transferred to a new tube and centrifuged at 4 °C, 10,000 × g for 45 min to remove large cellular debris and vesicles. The resulting supernatant was passed through a 0.22 μm filter, and the filtrate was collected. The filtrate was transferred to a new centrifuge tube and ultracentrifuged at 4 °C, 100,000 × g for 70 min using an ultracentrifuge rotor. The supernatant was discarded, and the pellet was resuspended in 10 mL of pre-chilled 1× PBS. Using the same rotor, a second round of ultracentrifugation was performed at 4 °C, 100,000 × g for 70 min. After discarding the supernatant, the pellet was resuspended in 200 μL of pre-chilled PBS. From this suspension, 20 μL was allocated for electron microscopy, 10 μL for particle size analysis, and 100 μL for protein extraction. The remaining exosomes were stored at −80 °C.

For transmission electron microscopy (TEM) imaging, 10 μL of the exosome suspension was applied onto a copper grid and allowed to settle for 1 min. Excess liquid was carefully removed using filter paper. Subsequently, 10 μL of uranyl acetate was applied to the grid for 1 min for negative staining, followed by removal of excess solution with filter paper. The grid was air-dried at room temperature for several minutes. Imaging was performed at 100 kV to obtain TEM results.

The diameter of the small sEVs was analyzed using nanoparticle tracking analysis (NTA). For each sample, five independent videos were captured with a camera level of 13 and a detection threshold of 5. All instrument acquisition parameters were fully recorded. Specific sEV markers were validated by Western blot.

### 2.22 ELISA

In order to determine the secretion of various inflammatory factors (IL-4, IL-5, IL-13, IL-33, CCL17, CCL22), HDM-specific IgE and total serum IgE before and after miR-146a-3p activation, ELISA kits (Mlbio, Shanghai, China) were used to detect them.

### 2.23 Public data acquisition and preprocessing

All public datasets used in this study were retrieved from the NCBI Gene Expression Omnibus (GEO). These included: GSE85567, bulk RNA-seq of human asthma (4 healthy controls vs. 4 asthma patients); GSE232805, bulk RNA-seq of NET-stimulated small airway epithelial cells (3 controls vs. 3 NET-treated samples); GSE227744, 10× Genomics scRNA-seq of BALF from asthma patients before and during acute exacerbation; and GSE297584, 10X Genomics H5 scRNA-seq of bronchial mucosal samples from 3 asthma patients (Asthma1–Asthma3; GSM8995180, GSM8995182, and GSM8995190) and 3 healthy controls (Control1–Control3; GSM8995184, GSM8995186, and GSM8995188). Importantly, in GSE227744, both “Before” and “During” samples were obtained from asthma patients and represent pre-exacerbation and exacerbation states, respectively. For bulk RNA-seq datasets, gene-level count matrices and the corresponding annotation file (Human.GRCh38.p13.annot.tsv.gz) were downloaded directly. Gene symbols were assigned according to the annotation file. When multiple transcripts corresponded to the same gene symbol, the transcript with the highest mean expression was retained. Entries lacking valid gene symbols were removed. Processed count matrices were converted to integers. Differential expression analysis was performed using DESeq2 at the sample level, with the criteria of |log_2_FC| > 1 and adjusted P < 0.05. Genes that failed independent filtering were retained as NA and were not used for significance calling.

### 2.24 miRNA validation in the asthma transcriptomic dataset

From the GSE85567 dataset, genes with gene symbols starting with "MIR" were extracted to construct a miRNA-related count matrix. After removing missing values, differential expression analysis was performed using DESeq2 with the same criteria as described above (|log_2_FC| > 1 and adjusted *P* < 0.05). For visualization, count data were transformed using the regularized logarithm (rlog) function, and a row-scaled heatmap was generated using the pheatmap package (v1.0.12) for five candidate miRNAs (MIR146A, MIR149, MIR18A, MIR191, and MIR203A), with sample conditions annotated in the color bar.

### 2.25 Single-cell RNA-seq analysis: neutrophil identification in exacerbation status (GSE227744)

GSE227744 contains 10× Genomics scRNA-seq data of BALF from asthma patients before and during acute exacerbation. Single-cell data were processed using the Seurat package (v4.3.0) in R (v4.5.2). Quality control retained cells with 200–6000 detected genes, 500–30,000 UMIs, mitochondrial reads <20%, and ribosomal reads <50%. Genes detected in at least three cells were included. Doublets were removed using scDblFinder (v1.16.0). After log-normalization, the top 2000 highly variable genes were used for PCA. Batch effects were then corrected using Harmony with sample origin (sample.id) as the batch variable. RunHarmony was applied in the PCA space with dims.use = 1:30. The Harmony embedding was used for UMAP dimensionality reduction, FindNeighbors, and FindClusters (resolution = 0.8). Clusters were annotated using canonical markers for T cells, B cells, NK cells, monocytes/macrophages, dendritic cells, neutrophils, and other stromal cells. Neutrophil markers included FCGR3B, CSF3R, S100A8, S100A9, CEACAM8, ITGAM, and CXCR2. The cluster with the highest average expression of these markers was defined as neutrophils.

### 2.26 *HIF1A* expression comparison and linear in silico knockdown (GSE227744)

*HIF1A* expression in neutrophils was compared between the "Before" and "During" states using the re-embedded UMAP and a Student's t-test. To assess the potential impact of *HIF1A* on neutrophil effector function, a linear perturbation model was applied to the "During" neutrophils subset. Cells expressing *HIF1A* above the median level and at least one of the target genes (ELANE or MPO) were selected for further analysis. The expression of each target gene was computationally reduced in proportion to the relative excess of *HIF1A* above the median, applying the formula: new_expr = max-(0,expr×(1−*HIF1A*excess*HIF1A*−median))new_expr = max(0,expr×(1−*HIF1A*−median*HIF1A*excess)). Pre- and post-knockdown expression levels were compared using paired Wilcoxon signed-rank tests. Results were visualized as box plots and a summary heatmap, with significance levels indicated by asterisks (*P < 0.05, **P < 0.01, ***P < 0.001).

### 2.27 Single-cell transcriptomic analysis of bronchial mucosa

Bronchial mucosal samples were obtained from GSE297584, including 3 asthma patients (Asthma1–Asthma3; GSM8995180, GSM8995182, and GSM8995190) and 3 healthy controls (Control1–Control3; GSM8995184, GSM8995186, and GSM8995188). Single-cell RNA sequencing data were acquired in 10X Genomics H5 format and analyzed using the Seurat package (v5). H5 files were read using Read10X_h5 to create Seurat objects. Quality control criteria included nFeature_RNA > 200, nCount_RNA > 500, mitochondrial gene percentage <25%, and ribosomal gene percentage <60%. Doublets were identified and removed using scDblFinder, followed by LogNormalize normalization and selection of 3000 highly variable genes for principal component analysis. Batch effects were corrected using Harmony with sample origin (sample) as the batch variable. RunHarmony was applied with dims.use = 1:30. The Harmony embedding was used for UMAP visualization and FindClusters clustering (resolution = 1.2). Cell types were annotated using SingleR with the HumanPrimaryCellAtlasData reference dataset, yielding cell types including T cells, B cells, NK cells, and myeloid cells. T cells were extracted for downstream analysis, and T cell counts were compared between groups using box plots. Module scores for Th2 and Th17 cells were calculated using AddModuleScore based on Th2 marker genes (GATA3, IL4, IL5, IL13, IL9, CCR4, CCR8, PTGDR2, IL1RL1, IL1R1, MAF, STAT6) and Th17 marker genes (RORC, RORA, IL17A, IL17F, IL17C, IL22, IL26, IL23R, IL21R, CCR6, KLRB1, BATF, STAT3), respectively. The 95th percentile of module scores was used as the threshold to define Th2 and Th17 cells (score above threshold and >0). Independent dimensionality reduction was performed for the defined Th2 and Th17 cells: normalization, selection of 1500 highly variable genes, scaling, PCA (30 PCs), FindNeighbors (dims = 1:15), FindClusters (resolution = 0.5), and UMAP (n.neighbors = 15, min.dist = 0.3, spread = 1). UMAP plots were generated to visualize the distribution of Th2 and Th17 cells, colored by group (asthma vs. control). The number and percentage of Th2 and Th17 cells per sample were calculated and displayed as box plots.

### 2.28 Flow cytometry

We extracted BALFs from mice for flow cytometry detection. After centrifugation of BALF at 3000 r for 5 min, discard the supernatant and add eBioscience Fixable Viability Dye eFluor 780 (65-0865-14, Thermo Fisher Scientific). After resuspending, incubate at 4 °C in the dark for 30 min. After the end of the incubation, add 1 ml of pre-chilled PBS and centrifuge again at 3000 r for 5 min. After discarding the supernatant, APC-conjugated CD45.2 antibody (#109814, Biolegend, CA, USA), PerCP-Cy5.5-conjugated CD3e antibody (45-0031-82, Thermo Fisher Scientific), and PE-conjugated SiglecF antibody (#155506, Biolegend) antibody were added and stained at 4 °C for 30 min in the dark. Wash again using pre-chilled PBS and centrifuge to discard the supernatant. Subsequently, 300 μL of pre-chilled PBS was added and the samples were detected using Cytoflex S (Beckman Coulter, Inc., Brea, CA, USA). Dimers and agglomerates were separated by setting up FSC-A with FSC-H, SSC-A with SSC-H, and analyzed using CytoExpert 2.4 software and CytoFLEX (A00-1-1102, Beckman Couter Biotechnology, Suzhou, China).

### 2.29 In vivo fluorescence tracking of miR-146a-3p-loaded exosomes

To enable in vivo tracking of miR-146a-3p, the miR-146a-3p-loaded exosome suspension was incubated with DiD working solution at 37 °C, after which unbound free DiD (II6234913, Lumiscience, Jilin, China) dye was removed by ultracentrifugation. The DiD-labeled, miR-146a-3p-carrying exosomes were then administered to mice via tail vein injection. In vivo fluorescence imaging was performed at 30 min, 1 h, 2 h, 4 h, 8 h, 16 h, 24 h, 48 h, and 72 h post-injection. Mice were maintained under continuous isoflurane anesthesia throughout the imaging sessions and positioned on the imaging platform. At each time point, the excitation/emission wavelengths, exposure time, and gain settings were kept constant across all animals to eliminate instrument-related variability. Whole-body fluorescence images were acquired to visually track the in vivo migration, tissue distribution, and metabolic clearance kinetics of the DiD-labeled miR-146a-3p exosomes. Regions of interest (ROIs) were delineated using the imaging system's accompanying software, and semi-quantitative analysis of fluorescence signals was performed based on the pseudo-color scale to evaluate the biodistribution and circulatory retention characteristics of the miR-146a-3p-loaded exosomes.

### 2.30 Statistical analysis

All statistical analyses were performed using Prism 7.0 (GraphPad) for experimental data and R (version 4.5.2) for bioinformatics and sequencing data. Data from in vitro experiments are presented as mean ± standard deviation (SD), and comparisons among multiple groups were evaluated using one-way analysis of variance (ANOVA) followed by Dunn's post-hoc test. For bioinformatics analyses, two-group comparisons were conducted using Wilcoxon rank-sum tests or Student's t-test, as appropriate. In all cases, *P* < 0.05 was considered statistically significant.

## 3 Results

### 3.1 Integrated Bioinformatic Analysis Implicates Neutrophil and ferroptosis in asthma pathogenesis

By integrating resources from Genecard, OMIM, and TTD databases, we systematically identified 9306 asthma-related targets. To focus on core regulatory molecules, we selected the top 2000 targets based on relevance to construct a protein-protein interaction (PPI) network (Fig. 1A). Topological analysis of this network subsequently identified 298 key pathogenic genes (Fig. 1B).

**Fig. 1:**
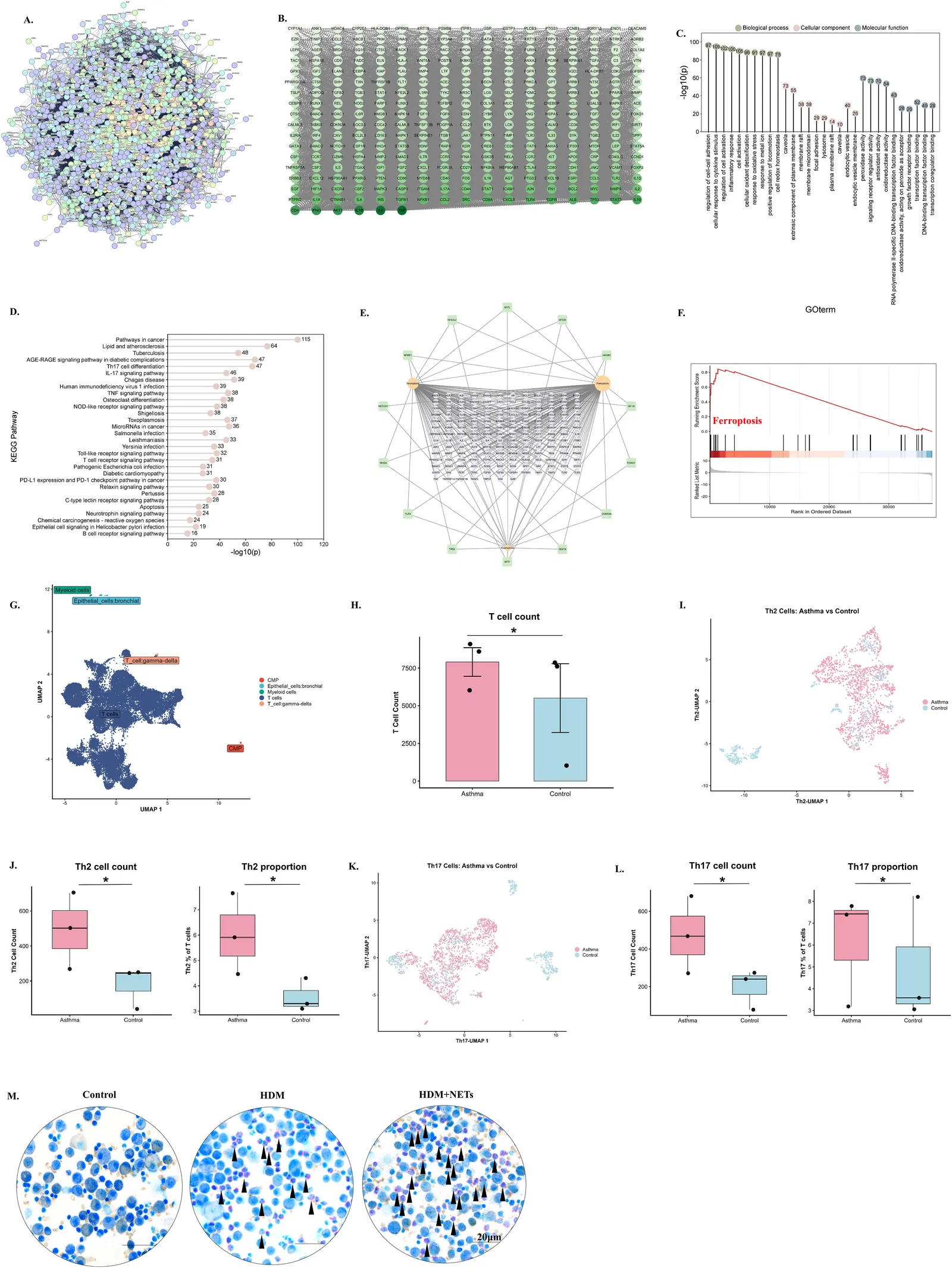
**Integrated Bioinformatic Analysis Implicates Neutrophil and ferroptosis in Asthma Pathogenesis (A)** Screening of asthma-related targets and construction of PPI network. **(B)** Topological analysis identifies key pathogenic genes in asthma. **(C)** GO functional enrichment analysis of key differentially expressed genes. **(D)** KEGG pathway enrichment analysis of differentially expressed genes. **(E)** Regulatory network of target genes and cell death patterns. **(F)** GSEA reveals significant enrichment of the ferroptosis pathway. **(G)** UMAP visualization of single-cell transcriptomes from human bronchial mucosal samples. Each dot represents an individual cell. **(H)** Comparison of captured T cell counts between asthmatic and control groups. Data are presented as mean ± SD (n = 3 per group). **(I)** UMAP visualization of re-clustered Th2 cell subpopulations, with cells colored by group: Asthma (pink) and Control (light blue). **(J)** Quantification of Th2 cell counts (left) and proportions among total T cells (right). Boxes represent the interquartile range (IQR) with the median line, and whiskers extend to the minimum and maximum (n = 3 per group). **(K)** UMAP visualization of re-clustered Th17 cell subpopulations. **(L)** Quantification of Th17 cell counts (left) and proportions among total T cells (right). Boxes represent the IQR, and whiskers extend to the minimum and maximum (n = 3 per group). Statistical comparisons were performed using an unpaired Student's t-test (or Mann-Whitney *U* test). UMAP, Uniform Manifold Approximation and Projection; SD, standard deviation; IQR, interquartile range. **(M)** Diff-quick staining images of BALF from mice in each group. Black arrows indicate neutrophils. Data are presented as mean ± SD (n = 3 for bioinformatics analyses in A–I, n = 6 per group for animal experiments in J). Scale bar = 20 μm *P < 0.05, **P < 0.01, ***P < 0.001.

To clarify the biological functions and regulatory pathways of these key genes, we further performed Gene Ontology (GO) enrichment analysis and KEGG pathway enrichment analysis (Fig. 1C and D). GO enrichment results indicated that these key genes were significantly enriched in biological processes, primarily involving the regulation of cell death, cell–cell interactions, and mediation of inflammatory responses. KEGG pathway analysis revealed that the target genes were most significantly enriched in the Th17 cell differentiation pathway and the IL-17 signaling pathway. Previous studies have confirmed that Th17 cells are the primary source of IL-17, which plays a key regulatory role in amplifying and sustaining neutrophil-mediated inflammatory responses [24], [25]. Combined with the GO analysis suggesting a close association between asthma pathogenesis and cellular damage, we speculate that neutrophil injury may further exacerbate the pathological progression of asthma by modulating related pathways. To explore the relationship between modes of cell death and core asthma targets, we compared public databases and performed intersection analysis of three prominent forms of cell death, including ferroptosis, cuproptosis, and necroptosis, with core asthma targets. The results showed the strongest association between asthma and ferroptosis, yielding 149 overlapping genes (Fig. 1E). Subsequent GSEA further confirmed that the ferroptosis pathway was significantly enriched in asthma samples (Fig. 1F).

While classical studies have predominantly focused on the Th2-dominant paradigm in asthma pathogenesis, our pathway enrichment analysis unexpectedly revealed a Th17-associated transcriptional signature that has not been fully elucidated by conventional stratification approaches. To dissect this distinct signal, we leveraged publicly available single-cell transcriptomic datasets derived from bronchial mucosal tissues of asthmatic and control individuals. UMAP-based clustering resolved major cellular lineages, with T lymphocytes dominating the adaptive immune compartment (Fig. 1G). This global architecture was accompanied by marked T-cell remodeling in asthmatic airways, reflected in increased total T-cell abundance relative to controls (Fig. 1H). We next interrogated the CD4^+^ T helper subsets that sustain asthmatic inflammation. It was revealed that profound rewiring of Th2 and Th17 programmes. Th2 cells showed increased absolute numbers and fractional representation within the T-cell pool, indicative of type 2 skewing (Fig. 1 I, J). Th17 populations were similarly expanded within the T-cell pool, suggesting concurrent type 17 engagement (Fig. 1 K, L). This coordinated enrichment implies a composite pathogenic state in which type 2 and type 17 programmes converge to shape the asthmatic inflammatory milieu. In vivo experiments further revealed significantly increased neutrophil counts in the BALF of HDM-challenged mice. Moreover, NETs further promoted neutrophil accumulation (Fig. 1. M).

In summary, our integrative analysis reveals an immune microenvironment imbalance characterized by Th17 and neutrophil enrichment, which coexists with the conventional Th2-dominant paradigm in asthma. Neutrophil-driven damage may facilitate asthma progression through ferroptosis-mediated inflammatory pathways.

### 3.2 NETs exacerbate symptoms in asthmatic mice and induce ferroptosis in lung tissue

NETs play a critical role in the pathogenesis of asthma and have been shown to drive the pathological process of asthma [26]. To clarify the role of NETs in HDM-induced allergic airway inflammation, this study first established an HDM-induced mouse model of allergic asthma (Fig. 2A). The results showed that compared with the control group, the levels of MPO-DNA complexes in both serum (Fig. 2B) and BALF (Fig. 2D) were significantly elevated in the HDM model group. Additionally, the relative expression of the NETs marker MPO-DNA in serum and BALF was also markedly upregulated (Fig. 2C–E), indicating substantial release of endogenous NETs in the HDM-induced asthma.

**Fig. 2:**
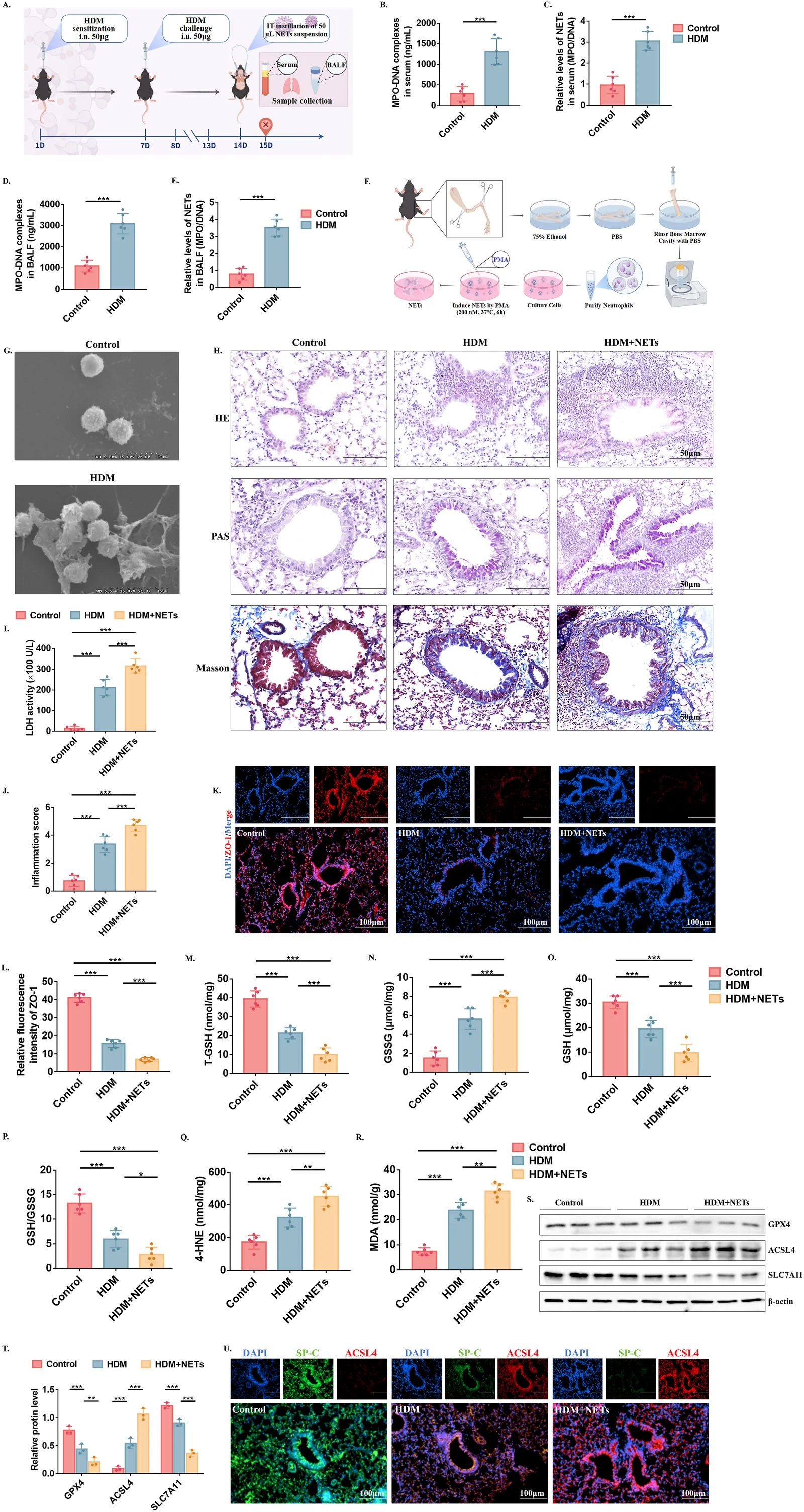
**NETs exacerbate symptoms in asthma mice and induce ferroptosis in lung tissue (A)** Experimental timeline for HDM-induced asthma mouse model and NETs intervention. **(B)** MPO-DNA complexes in serum. **(C)** The levels of NETs in serum. **(D)** MPO-DNA complexes in BLAF. **(E)** The levels of NETs in BALF. **(F)** Schematic illustration of the extraction for NETs derived from mouse bone marrow neutrophils. **(G)** SEM characterization of NETs. **(H)** Images of H&E-stained, periodic acid-Schiff-stained and Masson-stained lung tissue sections. Scale bar = 50 μm. **(I)** LDH levels in the serum of mice stimulated with NETs. **(J)** The inflammatory score was performed in a double-blind fashion. Comparison of LDH activity among different groups of mice. **(J)** Comparison of pulmonary inflammation scores among different groups of mice. **(K-L)** Immunofluorescence staining was performed to detect the expression and distribution of the tight junction core protein ZO-1 (red) in lung tissues of mice from each group. Nuclei were counterstained with DAPI (blue). Scale bar = 100 μm. **(M-P)** The levels of T-GSH, GSSG, GSH, and GSH/GSSG were measured by T-GSH/GSSG colorimetric assay kit. **(Q)** The level of 4-HNE were measured by 4-HNE ELISA kit. **(R)** The level of MDA were measured by MDA assay kit. **(S-T)** Western blotting analysis of ferroptosis-related protein levels in the lung tissue of mice. **(U)** Immunofluorescence staining were used to determine the localization of ACSL4 and SP-C. Scale bar = 100 μm. Green fluorescence represents alveolar epithelial cell marker protein SP-C, red fluorescence represents ferroptosis key protein ACSL4, and DAPI blue fluorescence represents the nucleus. Data are presented as mean ± SD (n = 6). *P < 0.05, **P < 0.01, ***P < 0.001.

To investigate the direct pathogenic role of NETs, we induced and purified structurally intact mouse neutrophil NETs in vitro (Fig. 2F–G) and administered them via intratracheal instillation to HDM-induced asthmatic mice. While HDM challenge alone induced moderate airway pathology, subsequent NETs treatment profoundly potentiated these effects, leading to a marked increase in inflammatory cell infiltration, goblet cell hyperplasia, and collagen deposition(Fig. 2H). Simultaneously, NETs further increased LDH activity in the BALF, suggesting aggravated airway epithelial cell injury (Fig. 2I). Semi-quantitative inflammation scores also confirmed their pro-inflammatory effects (Fig. 2J). Tight junctions are key structures maintaining the integrity of the airway epithelial barrier, and ZO-1, as a core molecule of the tight junction complex, is closely associated with barrier function [27]. We further detected the expression of ZO-1 in lung tissues across groups. Notably, a significant association was observed between NETs exposure and the downregulation of ZO-1, in the airway epithelium of asthmatic mice, suggesting that NETs may participate in regulating airway inflammation in asthma by disrupting the integrity of airway epithelial tight junctions (Fig. 2K and L).

Given that the inflammatory microenvironment is a key driver of specific forms of cell death, we further investigated whether NETs are involved in another type of cell death closely associated with oxidative stress—ferroptosis. After NETs intervention, the levels of total glutathione (T-GSH) and reduced glutathione (GSH) in the lung tissues of asthmatic mice were further decreased, while the content of oxidized glutathione (GSSG) was increased, leading to a significant reduction in the GSH/GSSG ratio (Fig. 2M–P). Meanwhile, the malondialdehyde (MDA) content and the positive signal intensity of 4-hydroxynonenal (4-HNE) were markedly enhanced compared with those in the asthmatic group without NETs intervention (Fig. 2Q and R). This indicates that NETs treatment further promotes the accumulation of lipid peroxidation products. Western blot analysis further confirmed that after NETs induction, the expression of the ferroptosis inhibitory proteins GPX4 and SLC7A11 in lung tissue was significantly downregulated, while the expression of ACSL4, a key regulatory protein of ferroptosis, was significantly upregulated (Fig. 2S and T). Consistently, immunofluorescence more directly showed that the number of ACSL4-positive cells was significantly increased in the lung tissue of asthmatic mice in the NETs intervention group (Fig. 2U). Collectively, these results indicate that substantial NETs release occurs in the HDM-induced asthma, and that exogenous NETs significantly exacerbate HDM-induced airway inflammation, epithelial injury, and airway remodeling. Furthermore, NETs promote the occurrence of airway epithelial ferroptosis by regulating the expression of key ferroptosis-related molecules.

### 3.3 NETs induce ferroptosis in BEAS-2B cells

To investigate the impact of NETs on the viability of BEAS-2B cells, we first treated the cells with varying concentrations of NETs (0, 100, 200, 400, and 800 ng/mL) extracted from neutrophils differentiated from HL-60. The CCK8 assay results revealed a concentration-dependent decrease in BEAS-2B cell viability with increasing NETs concentrations (Fig. 3A). Based on this concentration-response relationship and to ensure the effectiveness and consistency of subsequent experiments, we employed a concentration of 200 ng/mL NETs with a 12 h treatment duration for all subsequent in vitro assays.

**Fig. 3:**
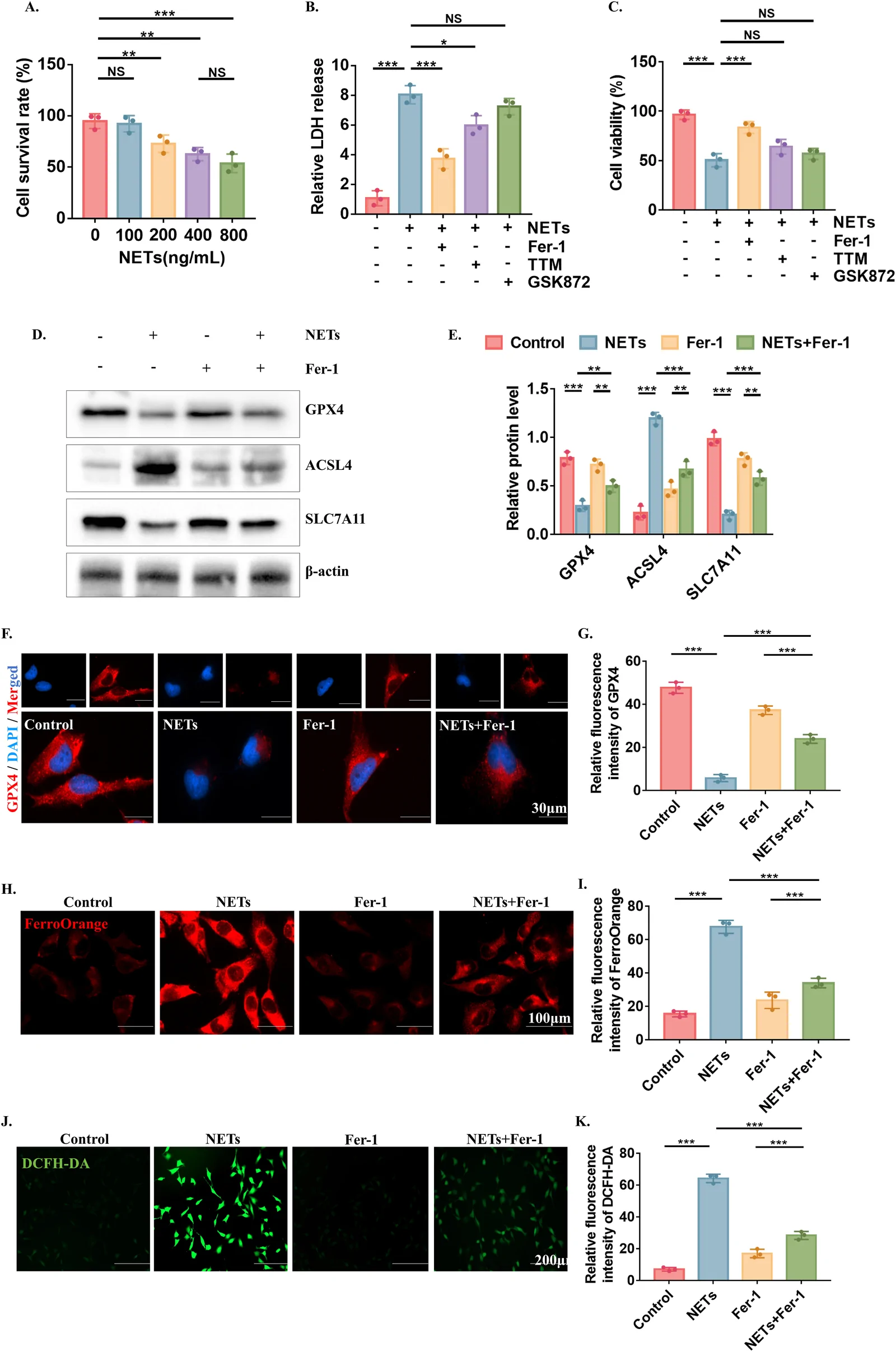
**NETs induce ferroptosis in BEAS-2B cells. (A)** CCK-8 assay. **(B)** The level of LDH in the supernatant of BEAS-2B cells was measured after treatment with ferroptosis, cuproptosis, and necroptosis inhibitors. **(C)** The cell survival of BEAS-2B cells was measured after treatment with ferroptosis, cuproptosis, and necroptosis inhibitors. **(D-E)** Western blotting analysis of ferroptosis-related protein levels in BEAS-2B. **(F-G)** The expression of GPX4 in BEAS-2B was detected by immunofluorescence. Scal bar = 30 μm. **(H-I)** The intracellular Fe^2+^ level was measured using the FerroOrange (red) fluorescent probe. Scal bal = 100 μm. **(J-K)** The ROS level was measured by using the DCFH-DA (green) probe. Scal bal = 100 μm. Data are presented as mean ± SD (n = 3). *P < 0.05, **P < 0.01, ***P < 0.001.

Building on prior analytical results, we observed that the ferroptosis signaling pathway was activated in NETs-treated BEAS-2B cells (Fig. 1E and F). To validate this finding, we introduced specific inhibitors, Ferrostatin-1 (a ferroptosis inhibitor), GSK872 (a necroptosis inhibitor), and TTM (a cuproptosis inhibitor), and compared their rescuing effects on NETs-mediated cellular damage. The LDH release assay results showed that NETs intervention significantly induced death-related injury in BEAS-2B. The ferroptosis-specific inhibitor Fer-1 markedly reversed NETs-mediated LDH release, whereas GSK872 and TTM exhibited no obvious cytoprotective effects (Fig. 3B). These findings suggest that bronchial epithelial cell injury induced by NETs is not dependent on the necroptosis or cuproptosis pathways. Cell viability assay results were consistent, showing that Fer-1 effectively restored the proliferative activity of BEAS-2B cells under NETs stimulation, while GSK872 and TTM intervention showed no significant improvement (Fig. 3C). These results confirm that ferroptosis is the core pathway mediating NETs-induced airway epithelial cell death. Further analysis revealed that NETs treatment significantly downregulated GPX4 and SLC7A11 expression while upregulating ACSL4 expression in BEAS-2B cells. Among these, GPX4 is a core inhibitory protein of ferroptosis. The upregulation of its expression, along with the expression changes of ACSL4 and SLC7A11, collectively confirms that NETs can induce cellular ferroptosis by regulating the expression of ferroptosis-related proteins. Importantly, Ferrostatin-1 pretreatment effectively reversed these aberrant expression patterns (Fig. 3D and E). This conclusion was further corroborated by immunofluorescence staining results (Fig. 3F and G). Given that the hallmark of ferroptosis is iron-dependent lipid peroxidation, we next assessed intracellular Fe^2+^ release and reactive oxygen species (ROS) generation. The results indicated that NETs treatment significantly increased Fe^2+^ release in BEAS-2B cells, whereas Ferrostatin-1 pretreatment notably reduced Fe^2+^ levels (Fig. 3H and I). Concurrently, NETs treatment promoted increased ROS production, and Fer-1 intervention effectively suppressed this excessive ROS generation (Fig. 3J and K).

Collectively, these in vitro experimental findings demonstrate that NETs play a crucial role in inducing ferroptosis in BEAS-2B cells by modulating the expression of key ferroptosis-related molecules, promoting Fe^2+^ accumulation, and driving excessive ROS production.

### 3.4 Activation of the cGAS-STING pathway by NETs contributes to ferroptosis

The specific structures released upon neutrophil activation, NETs, and their intersecting regulatory network with ferroptosis, in asthma remain incompletely elucidated. To uncover their intrinsic connection, this study employed integrated multi-database analysis. First, we identified 1193 common targets between NETs and the ferroptosis pathway (Fig. 4A), suggesting a potentially close regulatory network mediated by core targets. To clarify the functional enrichment characteristics of these common targets, we performed KEGG enrichment analysis on the intersecting targets. The results showed that the Cytosolic DNA sensing pathway, a key signaling pathway that detects abnormal DNA in the cytoplasm and initiates innate immune responses, among which the cGAS-STING pathway is the most in-depth studied core component (Fig. 4B). The cGAS-STING pathway, a core component of innate immunity, can initiate downstream inflammatory responses and cell death signals by recognizing cytosolic nucleic acids. Previous studies have confirmed abnormal activation of this pathway in inflammation-related diseases such as asthma and autoimmune diseases [28], [29], providing a critical direction for exploring the regulatory mechanisms between NETs and ferroptosis.

**Fig. 4:**
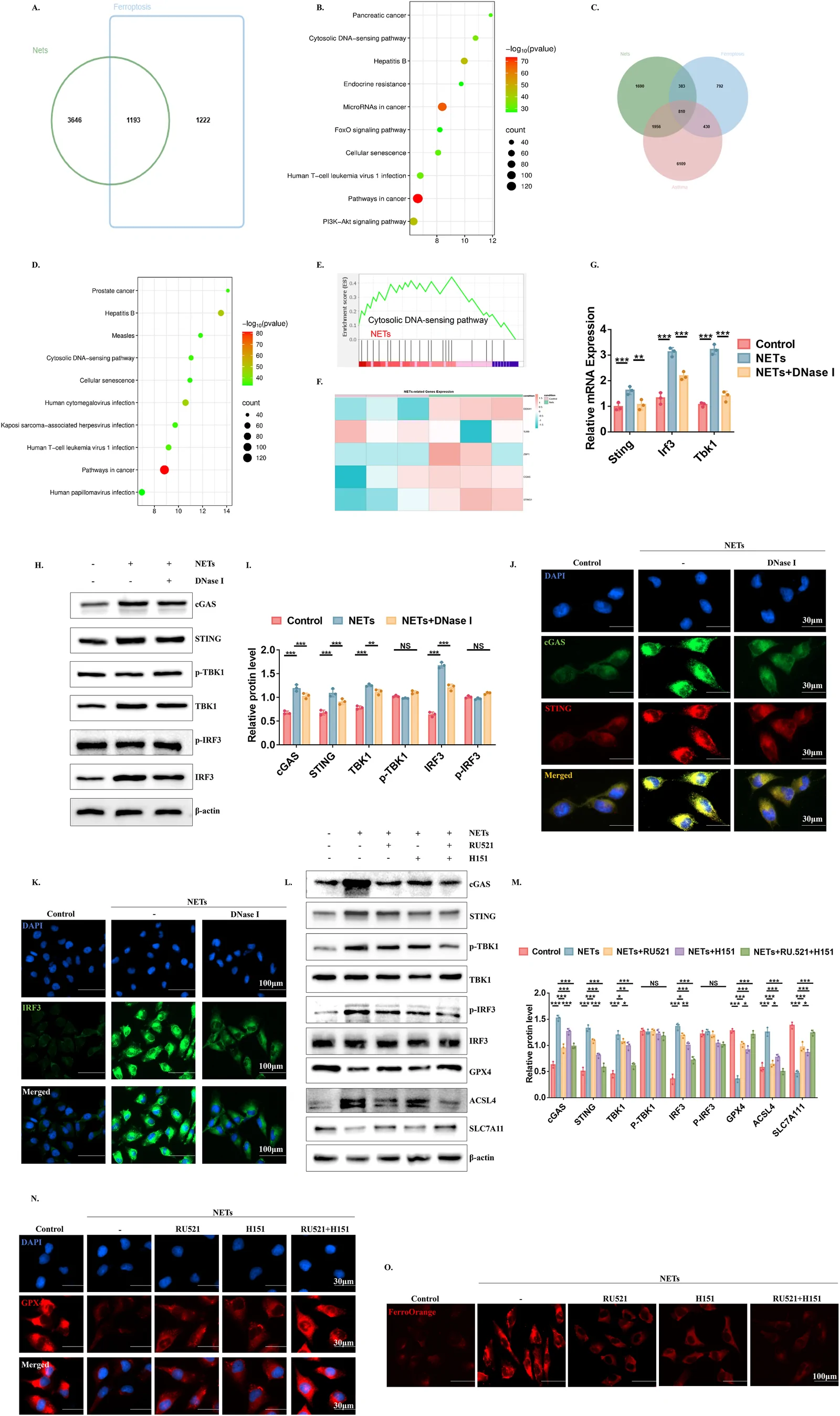
**Activation of the cGAS-STING pathway by NETs contributes to ferroptosis**. **(A)** Venn diagram of genes related to NETs and ferroptosis. **(B)** Bubble diagram of KEGG pathway enrichment for target genes. **(C)** Venn diagram of genes related to NETs, ferroptosis and asthma. **(D)** Bubble plot of KEGG pathway enrichment for target genes. **(E)** GSEA enrichment plot of the Cytosolic DNA-sensing pathway in the NETs gene set. **(F)** Heatmap of NETs-related gene expression.**(G)** RT-qPCR was performed to analyze the mRNA expression levels of *STING*, *IRF3* and *TBK1*. **(H-I)** Western blotting analysis of cGAS-STING pathway protein levels in BEAS-2B. **(J)** The expression of cGAS (green) and STING (red) in BEAS-2B cells was detected by immunofluorescence. Nuclei were counterstained with DAPI (blue). Scal bal = 30 μm. **(K)** The expression of IRF3 (green) in BEAS-2B was detected by immunofluorescence. Nuclei were counterstained with DAPI (blue). Scal bal = 100 μm. **(L-M)** Western blotting analysis of ferroptosis-related protein and cGAS-STING pathway protein levels in BEAS-2B cells was performed. **(N)** The expression of GPX4 (red) in BEAS-2B was detected by immunofluorescence. Nuclei were counterstained with DAPI (blue). **(O)** The intracellular Fe^2+^ level was measured using the FerroOrange (red) fluorescent probe. Scal bal = 100 μm. Scal bal = 30 μm. Data are presented as mean ± SD (n = 3). *P < 0.05, **P < 0.01, ***P < 0.001.

We further conducted a three-way intersection analysis of NETs-related genes, ferroptosis-related genes, and asthma disease genes, obtaining 810 shared targets (Fig. 4C). This suggests potential synergistic regulatory roles among these three factors in asthma pathogenesis. Re-screening of this three-way intersection target set via KEGG analysis reconfirmed significant enrichment in the cGAS-STING pathway (Fig. 4D), further reinforcing the potential role of the cGAS-STING pathway as a core regulatory hub for these three elements. To confirm the activation status of the cGAS-STING pathway in asthma, we performed GSEA, which revealed a significantly activated phenotype of this pathway in asthma samples (Fig. 4E), consistent with previous findings that "aberrant activation of the cGAS-STING pathway can exacerbate airway inflammation and asthma pathological injury [29]". Bioinformatics analysis of the GEO database further confirmed aberrant expression of cGAS-STING pathway-associated genes in BEAS-2B cells. Compared with the normal control group, treatment with NETs significantly altered the expression levels of cGAS-STING pathway-associated genes in BEAS-2B cells (Fig. 4F), indicating that NETs can regulate the activation status of the cGAS-STING pathway.

To define the involvement of NETs in ferroptosis and cGAS-STING signaling, intervention experiments were performed with DNase I, a specific NET-degrading agent. DNase I effectively reversed the NETs-induced aberrant expression of ferroptosis-related marker proteins (Fig. S1A and B). On this basis, RT-qPCR was used to assess whether NETs modulate the cGAS-STING pathway. In NETs-treated BEAS-2B cells, the key cGAS-STING genes *Sting*, *Tbk1*, and *Irf3* were significantly upregulated, whereas DNase I abolished this activation (Fig. 4G), indicating that NETs may amplify inflammatory responses through cGAS-STING signaling. DNase I similarly counteracted the NETs-driven increase in total protein level (Fig. 4H and I). Because TBK1 and IRF3 phosphorylation is a critical step for downstream interferon production in the cGAS-STING axis [30], the dissociation between total protein levels and phosphorylation status here implies that NETs may regulate this pathway and promote ferroptosis primarily by altering the abundance of core cGAS-STING components rather than their phosphorylation. To delineate downstream cGAS-STING activation, immunofluorescence was performed. NETs stimulation elicited perinuclear redistribution of STING in Beas-2B cells and increased total IRF3 expression with mild nuclear accumulation (Fig. 4J and K). Since perinuclear translocation of STING is essential for its downstream signal transduction, these morphological findings support the notion that NETs activate cGAS-STING signaling via STING perinuclear redistribution and IRF3 nuclear accumulation, providing a basis for subsequent mechanistic validation. To determine how cGAS-STING signaling contributes specifically to NETs-triggered ferroptosis in BEAS-2B cells, the cGAS inhibitor RU.521 and the STING inhibitor H-151 were used. Immunoblotting showed that both inhibitors substantially reversed the NETs-induced dysregulation of GPX4, ACSL4, and SLC7A11 (Fig. 4L, M), a finding supported by immunofluorescence (Fig. 4N). In parallel, intracellular iron measurement revealed that RU.521 and H-151 markedly attenuated NETs-evoked iron accumulation (Fig. 4O). Given that intracellular iron overload is a central initiating event in ferroptosis, these data link cGAS-STING signaling to NETs-triggered ferroptosis from the perspective of iron metabolism.

In summary, this study demonstrates through multi-faceted experiments that NETs can induce ferroptosis in Beas-2B cells by activating the cGAS-STING pathway, thereby regulating ferroptosis-related protein expression and intracellular iron homeostasis. Inhibiting the cGAS-STING pathway can effectively block the NETs-mediated ferroptosis process in vitro.

### 3.5 miR-146a-3p exerts its regulatory effect by targeting HIF-1α

MicroRNAs (miRNAs), as central molecules in the post-transcriptional regulatory network of gene expression, can specifically target downstream genes and extensively participate in various critical biological processes such as inflammatory responses [31], ferroptosis [32], and signal pathway activation. Our previous studies have found that the miR-146a family exhibits aberrant expression patterns in asthma [33] and inflammation-related diseases [34] (Fig. 5A). Meanwhile, analysis of human asthma transcriptome data from the NCBI database revealed that miR-146a (MIR146A) expression was significantly lower in asthma patient samples compared with healthy controls, displaying an asthma-specific downregulation pattern (Fig. 5B). Among them, the function of miR-146a-5p, a member of this family, has been clearly demonstrated to be involved in regulating the pathogenesis of asthma [35]. In light of this, to further refine the inflammation regulatory network mediated by NETs and clarify its intrinsic relationship with miRNA regulatory pathways, this study focuses on elucidating the downstream molecular regulatory mechanisms of miR-146a-3p. Based on the differentially expressed miRNAs, we constructed a regulatory network of miRNA downstream targets (Fig. 5C) and conducted GO/KEGG enrichment analysis on the downstream target genes. The differentially expressed target genes were primarily enriched in biological processes including transcriptional regulation, nucleocytoplasmic transport, and cell cycle progression (Fig. 5D and E), suggesting that these miRNAs may contribute to asthma-related pathogenesis by modulating key transcription factors. Nucleocytoplasmic transport is a critical intracellular process that governs material trafficking and signal transduction; its dysfunction can affect downstream gene transcription, protein localization, and signaling pathway activation, thereby regulating inflammatory responses and cell death processes [36]. To further identify core regulatory nodes, we performed a Venn analysis integrating predicted miRNA target genes, transcription factors, and disease-associated genes, which revealed *HIF1A* and *RB1* as potential core target genes of miR-146a-3p (Fig. 5F). However, based on a comprehensive assessment of target binding efficiency, HIF-1α exhibited the most prominent binding activity and was consequently identified as the core target gene of this study. HIF-1α, a core member of the hypoxia-inducible factor family, not only regulates the hypoxic stress response but has been shown to modulate inflammatory responses [37]. The potential link between HIF-1α and both NETs and ferroptosis has thus emerged as a central focus for future investigations. To verify the binding specificity between miR-146a-3p and the core targets, further analysis revealed that the binding affinity between miR-146a-3p and *HIF1A* was significantly higher than that with *RB1*, and seven potential binding sites were co-identified (Fig. 5G), providing molecular structural evidence for a direct regulatory relationship between the two. Furthermore, to assess the evolutionary conservation and biological stability of the binding sites, we performed conservation analysis on the miR-146a-3p binding region on *HIF1A* (Fig. 5H). The results indicated that this binding site is highly conserved across species, further supporting the biological significance of miR-146a-3p in regulating *HIF1A* expression and laying the groundwork for subsequent in vitro functional validation experiments. Dual-luciferase reporter assay results further confirmed that miR-146a-3p can directly bind to the 3'-UTR region of *HIF1A* and significantly inhibit its gene expression activity through binding to specific sites. When these binding sites were mutated, the inhibitory effect of miR-146a-3p completely disappeared, demonstrating strict sequence specificity in their interaction (Fig. 5I). Subsequent functional validation experiments yielded consistent results, showing that miR-146a-3p could significantly downregulate both the mRNA and protein expression levels of *HIF1A* (HIF-1α) (Fig. 5J–L).

**Fig. 5:**
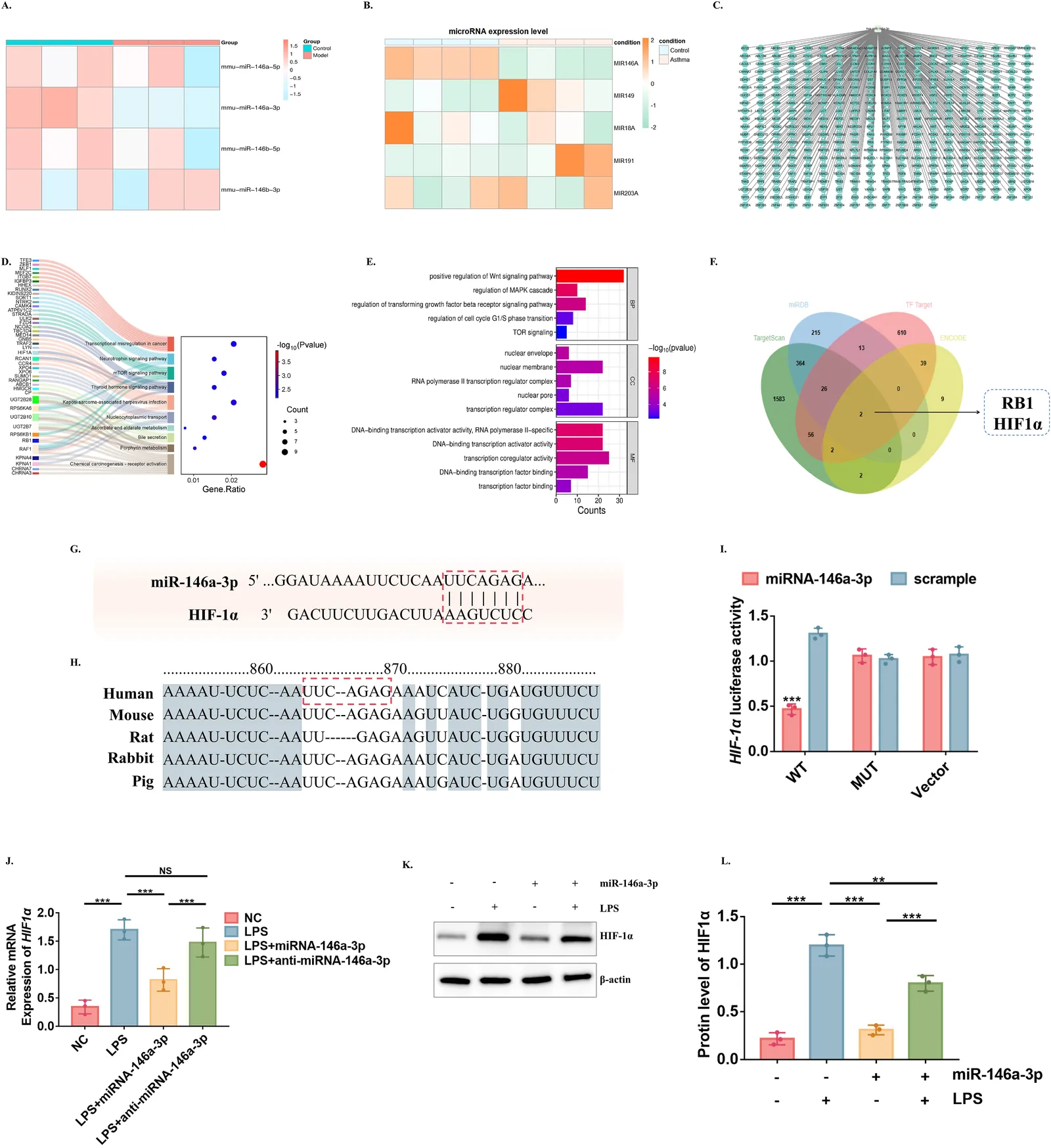
**miR-146a-3p exerts its regulatory effect by targeting HIF-1α (A)** Heatmap of miR-146 family microRNA expression in the control and model groups based on transcriptomic analysis. **(B)** Heatmap of candidate microRNA expression in control and asthma groups based on public database analysis. **(C)** Target network of miR-146a-3p. **(D)** Sankey diagram of KEGG pathway enrichment of target genes. **(E)** GO functional enrichment analysis of target genes. **(F)** Venn diagram of multi-database intersection for screening downstream transcription factors of miR-146a-3p. **(G)** Schematic diagram of predicted binding site between miR-146a-3p and HIF-1α. **(H)** Sequence conservation alignment of the miR-146a-3p binding region in the HIF-1α 3'UTR across multiple species. **(I)** Dual-luciferase reporter assay validation of miR-146a-3p targeting HIF-1α. **(J)** qRT-PCR analysis of *HIF1α* mRNA expression. **(K-L)** Western blotting analysis of HIF-1α protein levels. Data are presented as mean ± SD (n = 3). *P < 0.05, **P < 0.01, ***P < 0.001.

The above results preliminarily reveal the regulatory characteristics of miR-146a-3p targeting HIF-1α as a core. miR-146a-3p likely controls HIF-1α activity through regulating its nucleocytoplasmic shuttling and transcriptional function, providing new insights into the pathogenesis of inflammatory disorders.

### 3.6 HIF-1α mediates the generation of NETs

Using publicly available single-cell transcriptomic data from asthma patients, we first performed clustering analysis on all captured cells and annotated the resulting clusters on the UMAP plot using SingleR, identifying a total of 14 cell clusters (Fig. S1A–B). Focusing on neutrophils, we validated the identity of the neutrophil cluster using a well-established set of neutrophil signature genes, including *FCGR3B, CSF3R, S100A8, S100A9, CEACAM8, ITGAM*, and *CXCR2* (Fig. S1C–G). We then compared the HIF-1α expression profiles between purified neutrophil subpopulations obtained during the pre-inflammatory phase (Before, n = 2029) and those obtained during acute exacerbation (During, n = 5263) (Fig. 6A). UMAP feature plots revealed that HIF-1α was broadly expressed at low levels in neutrophils during the pre-inflammatory phase, with only a few cells displaying moderate signals. In contrast, neutrophils from the acute exacerbation phase exhibited markedly elevated HIF-1α expression, accompanied by a pronounced increase in the proportion of cells with high expression levels. Visualization of the combined dataset further confirmed that the acute exacerbation status of asthma strongly induced HIF-1αupregulation in neutrophils, suggesting that HIF-1α plays a critical role in orchestrating functional reprogramming of neutrophils and driving asthma pathogenesis.

**Fig. 6:**
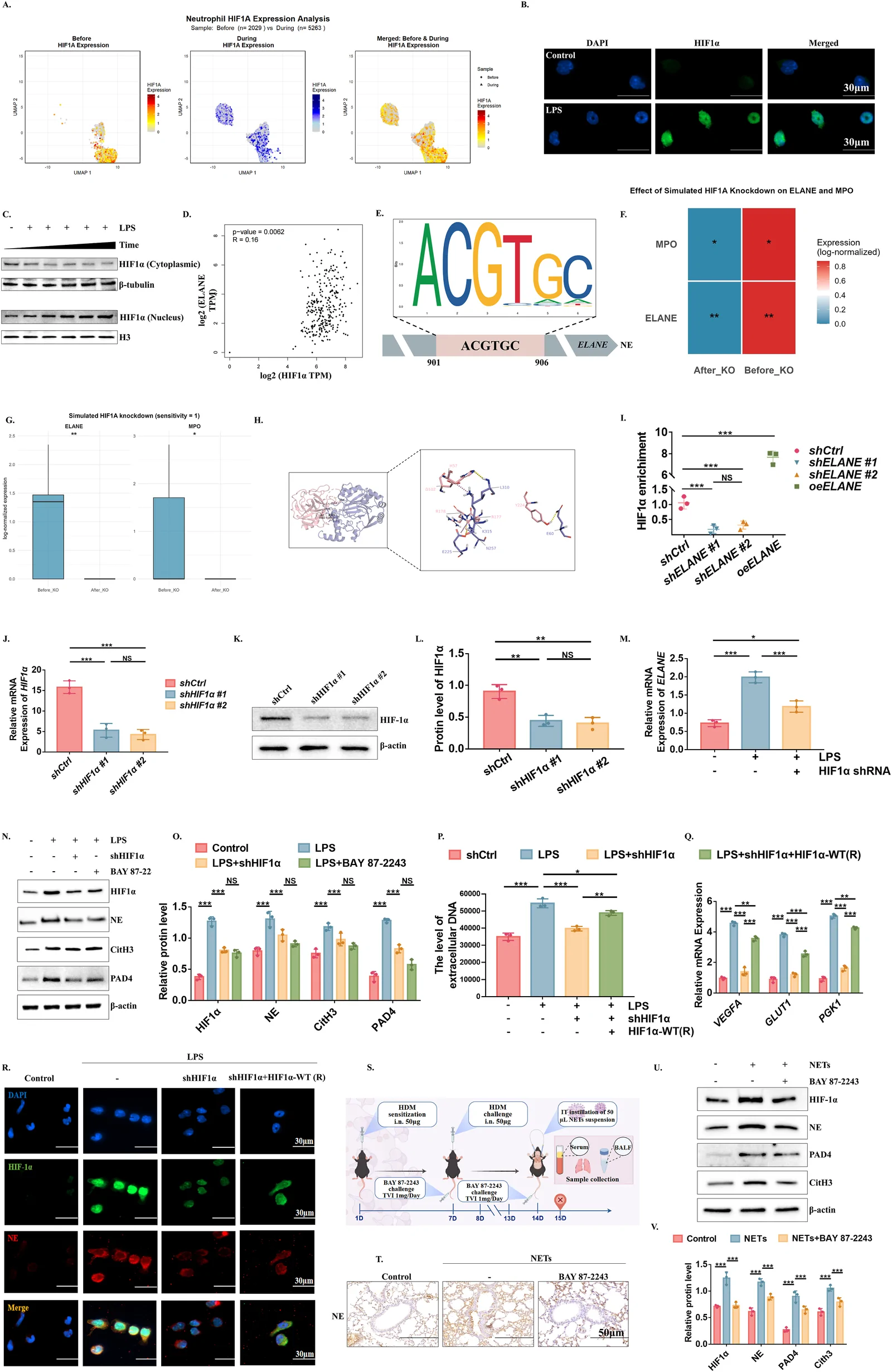
**HIF-1α mediates the generation of NETs. (A)** UMAP visualization of HIF-1α expression in neutrophils before and during asthma onset. **(B)** Immunofluorescence analysis demonstrated that LPS treatment induced the nuclear translocation of HIF-1α. DAPI staining shows the nuclei (blue), the HIF-1α fluorescence signal is green, and the Merge panel shows the superimposed images. Scale bar = 30 μm. **(C)** Time-dependent effects of LPS treatment on HIF-1α protein expression and nuclear localization. **(D)** Correlation analysis of *HIF1A* and *ELANE* gene expression. R = 0.16, *p* = 0.0062. **(E)** Analysis of the binding motif for HIF-1α in the *ELANE* promoter region. **(F)** Linear simulation of *ELANE* and *MPO* expression changes in the *HIF1A* knockdown model. **(G)** Box plots showing log-normalized expression of *ELANE* and *MPO* before and after simulated *HIF1A* knockdown (sensitivity = 1). **(H)** Schematic diagram of the docking between HIF-1α and NE protein. **(I)** Analysis of changes in HIF-1α binding enrichment at the target sequence by ChIP-qPCR. **(J)** Detection of mRNA expression levels after *HIF1A* interference by RT-qPCR. **(K-L)** Detection of protein levels after HIF-1α interference by Western blot. **(M)** Effect of HIF-1α knockdown on *ELANE* mRNA expression. **(N-O)** Effect of HIF-1α regulation on the expression of NETs-related proteins. **(P)** The regulatory role of HIF-1α on extracellular DNA release. **(Q)** Regulatory analysis of sh*HIF1A* interference and HIF-1α rescue on the mRNA expression of its downstream target genes. **(R)** Immunohistochemical analysis of the effect of HIF-1α inhibition on LPS-induced NE expression in mouse lung tissue. **(S-T)** Regulation of HIF-1α inhibitor on the expression of LPS-induced NETs-related proteins in mouse lung tissue. **(U)** The expression of MPO and CitH3 in dHL-60 cells was detected by immunofluorescence. Scal bal = 30 μm. Data are presented as mean ± SD (n = 3 per group for cell experiments in A-Q, n = 6 per group for animal experiments in R-U). *P < 0.05, **P < 0.01, ***P < 0.001.

Nuclear translocation of HIF-1α is prerequisite for its transcriptional activity. It is well established that HIF-1α must enter the nucleus, form a functional heterodimer with HIF-1β, and bind to the hypoxia response elements (HREs) in the promoters of target genes to exert its regulatory functions [38]. We therefore assessed its subcellular localization. Immunofluorescence staining confirmed its progressive enrichment in the nucleus upon LPS challenge (Fig. 6B). Consistently, western blot analysis of nuclear and cytoplasmic fractions revealed that LPS stimulation induced a time-dependent accumulation of HIF-1α in the nuclear compartment, which peaked at 4-6 h (Fig. 6C). In contrast, the levels of the cytoplasmic marker β-tubulin remained exclusively in the cytoplasmic fraction, confirming the purity of the separation.

NE, encoded by *ELANE*, is a core effector molecule in the inflammatory microenvironment mediated by NETs [39]. Dysregulated *ELANE* expression has been repeatedly linked to the HIF-1α-mediated inflammatory regulatory network in models such as ARDS [40]. Having confirmed the nuclear transcriptional activity of *HIF1A*, we further investigated its functional link with *ELANE*. Gene expression correlation analysis showed a significant positive correlation between the log_2_ (TPM) expression levels of *ELANE* and *HIF1A* (R = 0.16, *p* = 0.0062, Fig. 6D), suggesting a potential regulatory connection. Motif analysis at the sequence level provided a molecular target for this link: within the hexameric sequence ACGTGC at positions 901-906 upstream of the *ELANE* gene, the information entropy of the first three bases (A, C, G) was significantly high, indicating strong evolutionary conservation in this region and identifying it as a potential core transcriptional regulatory element (Fig. 6E). The presence of this highly conserved motif provides a direct sequence target for HIF-1α-mediated transcriptional regulation of *ELANE*, implying that HIF-1α may regulate *ELANE* transcription by binding to this motif, thereby establishing a molecular basis for their functional association. Following additional knockdown of *HIF1A* expression via linear simulation, NE exhibited a more significant decrease compared with MPO, implying a potentially greater regulatory role of HIF-1α in modulating NE (Fig. 6F and G). To further verify the structural feasibility of this regulatory interaction, we analyzed the interaction pattern between HIF-1α and NE via protein docking. The binding interface revealed a stable intermolecular interaction network formed by residues such as H57, D102, and L310 through hydrogen bonding, hydrophobic stacking, and other forces (Fig. 6H). ChIP-qPCR experiments directly confirmed the targeting of this binding, as the results demonstrated a significant increase in HIF-1α enrichment at the *ELANE* upstream regulatory region in the *ELANE* overexpression group, while showing a marked reduction in the *ELANE* knockdown groups (sh*ELANE* #1 and #2) (Fig. 6I). Notably, there was no significant difference (NS) between the two sh*ELANE* groups. This result provides direct evidence for the targeted binding of HIF-1α to the *ELANE* regulatory region.

To delve deeper into the regulatory mechanism of HIF-1α on NE transcription, after validating the efficiency of HIF-1α knockdown (Fig. 6J–L), we examined its impact on NE mRNA and protein expression levels. The results showed that HIF-1α knockdown significantly inhibited NE mRNA expression(Fig. 6I). Concurrently, HIF-1α knockdown markedly suppressed the LPS-induced protein expression of NETs-related components, with an inhibitory effect comparable to that of the specific HIF-1α inhibitor BAY 87-2243 (Fig. 6N and O). Extracellular free DNA is a key hallmark of NETs formation. To investigate the effect of HIF-1α on NETs generation, we performed rescue experiments. We found that HIF-1α knockdown significantly reduced the LPS-induced increase in extracellular free DNA. Notably, the NETs-forming capacity was restored in the knockdown cells upon rescue with an shRNA-resistant wild-type HIF-1α (Fig. 6P). More importantly, the expression of HIF-1αdownstream target genes such as *VEGFA*, *GLUT1*, and *PGK1* was partially restored following rescue, directly proving the transcriptional activity of the rescued HIF-1α (Fig. 6Q). Immunofluorescence provided more intuitive evidence for the regulatory role of HIF-1α on key NETs components (Fig. 6R). In vivo validation experiments further demonstrated that immunohistochemical results indicated BAY 87-2243 significantly inhibited NE expression (Fig. 6S and T) and the expression of NETs-related proteins (Fig. 6U and V) in lung tissues of asthmatic mice.

In summary, from the foundational transcriptional activity based on subcellular localization, to the correlative trends in gene expression, and further to the molecular basis of sequence targeting and the structural support from protein interactions, multi-faceted evidence collectively constructs a complete regulatory chain for the functional association between HIF-1α and NE.

### 3.7 Efficient delivery of miR-146a-3p to neutrophils is achieved by BEAS-2B exosomes

Emerging evidence has revealed that the BEAS-2B is capable of secreting abundant exosomes, which serve as critical vehicles for intercellular communication by transferring signaling molecules [41]. Given the pivotal role of airway epithelial cells in orchestrating immune responses in neutrophilic asthma, we hypothesized that BEAS-2B-derived exosomes might exert a negative regulatory effect on neutrophilic inflammation.

To test this, we isolated exosomes from the conditioned medium of BEAS-2B cells using a standard ultracentrifugation protocol, a gold standard method widely employed for exosome purification due to its high efficiency in enriching intact extracellular vesicles (EVs) (Fig. 7A). The isolated vesicles were characterized using multiple approaches. Transmission electron microscopy (TEM) revealed that these BEAS-2B-derived EVs (BEAS-2B-EVs) exhibited a typical spherical morphology with a distinct bilayer membrane structure, a hallmark feature of exosomes (Fig. 7B). Nanoparticle tracking analysis (NTA) further demonstrated that the vesicles had a mean particle size of 181.6 nm (Fig. 7C and D), which falls within the canonical size range (30–200 nm) of exosomes. In addition, Western blot analysis confirmed the presence of exosome-specific surface markers, including CD63, CD9, CD81 and TSG101, which were highly enriched in the isolated EV fraction (Fig. 7E). Collectively, these morphological, size-distribution and molecular characterization data unequivocally validated that the purified particles were bona fide extracellular vesicles. Subsequently, we established BEAS-2B cell models with either overexpression or inhibition of miR-146a-3p and collected the exosomes secreted by these cells. qPCR analysis revealed that miR-146a-3p levels were markedly upregulated in exosomes derived from BEAS-2B cells overexpressing agomiR-146a-3p, whereas exosomes from cells transfected with miR-146a-3p-inhabitor showed significantly reduced miR-146a-3p expression (Fig. 7F), indicating that exosomes efficiently carry and deliver epithelium-derived miR-146a-3p. To investigate the anti-inflammatory function of exosomal miR-146a-3p, we performed intervention experiments in an LPS-induced inflammation model. LPS stimulation significantly induced the release of the inflammatory cytokines IL-33, CCL17, and CCL22 from neutrophils. Treatment with exosomes overexpressing miR-146a-3p markedly suppressed the secretion of these cytokines. In contrast, exosomes with miR-146a-3p inhibition failed to exert an obvious anti-inflammatory effect and instead further exacerbated the LPS-induced inflammatory response (Fig. 7G–I). Taken together, these findings demonstrate that BEAS-2B cell-derived exosomes serve as effective delivery vehicles for miR-146a-3p, transporting it to neutrophils and exerting robust anti-inflammatory effects, and that this protective effect is primarily dependent on the functional miR-146a-3p carried by the exosomes.

**Fig. 7:**
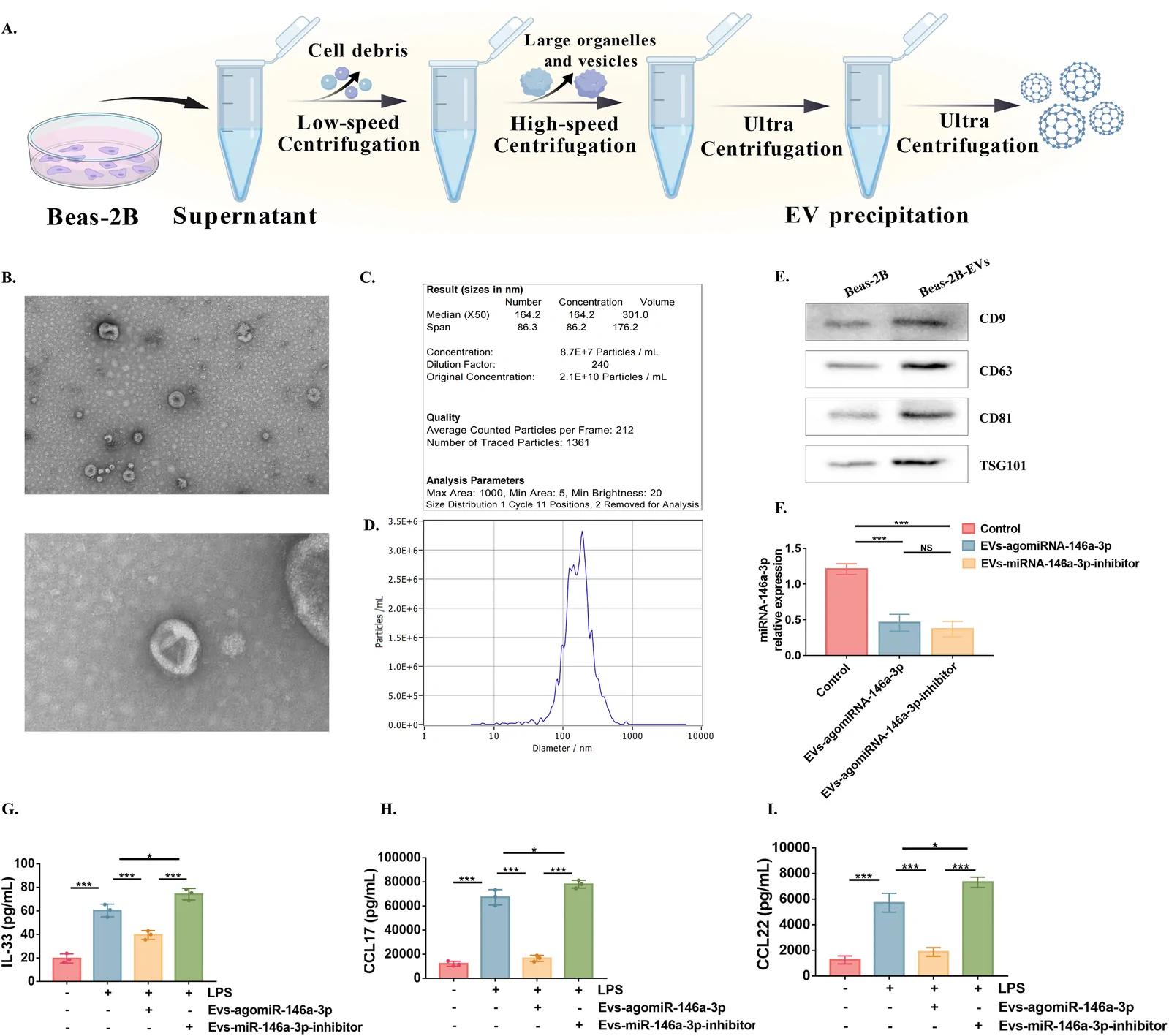
**Efficient delivery of miR-146a-3p to neutrophils is achieved by BEAS-2B exosomes**. **(A)** Procedure for the extraction of exosomes derived from BEAS-2B cells. **(B)** BEAS-2B of sEVs determined by TEM. **(C-D)** Diameter and concentration of sEVs examined by NTA. **(E)** Detection of exosomal marker protein expression levels by Western blot. **(F)** Detection of miR-146a-3p interference efficiency by qPCR. **(G-I)** Detection of expression levels of related inflammatory factors by ELISA. Data are presented as mean ± SD (n = 3) *P < 0.05, **P < 0.01, ***P < 0.001.

### 3.8 *Exosomal miR-146a-3p from BEAS-2B cells* inhibits *NET formation and alleviates ferroptosis by targeting HIF-1α*

To investigate the functional role of BEAS-2B exosomal miR-146a-3p, we first examined its regulatory effect on NETs formation in an LPS-induced neutrophil inflammation model. Exosomes overexpressing miR-146a-3p significantly reversed LPS-induced excessive NET release (Fig. 8A–C). Immunofluorescence provided more intuitive evidence that NET-associated fluorescence was considerably weaker in the EVs-agomiR-146a-3p group than in LPS-stimulated controls (Fig. 8D), supporting an inhibitory role for miR-146a-3p in NETs formation. To define the molecular dependency of this effect, we focused on TSG101, an ESCRT component required for exosome biogenesis or secretion and a key mediator of miRNA cargo sorting [42]. Specifically, TSG101 was knocked down or overexpressed in BEAS-2B donor cells before exosome isolation. RT-qPCR showed that exosomal miR-146a-3p levels were markedly reduced in the sh-TSG101 group compared with the sh-NC group (Fig. S3A), whereas intracellular miR-146a-3p levels remained largely unchanged (Fig. S3B), indicating that TSG101 mainly affects miR-146a-3p loading into exosomes rather than its cellular expression. The purified exosomes were then applied to LPS-stimulated neutrophils. Under these conditions, TSG101 knockdown in donor cells reversed the miR-146a-3p-mediated suppression of NET formation (Fig. 8E and F), whereas TSG101 overexpression enhanced this inhibitory effect (Fig. 8G and H). These indicate that exosomal delivery is required for miR-146a-3p to exert its biological activity.

**Fig. 8:**
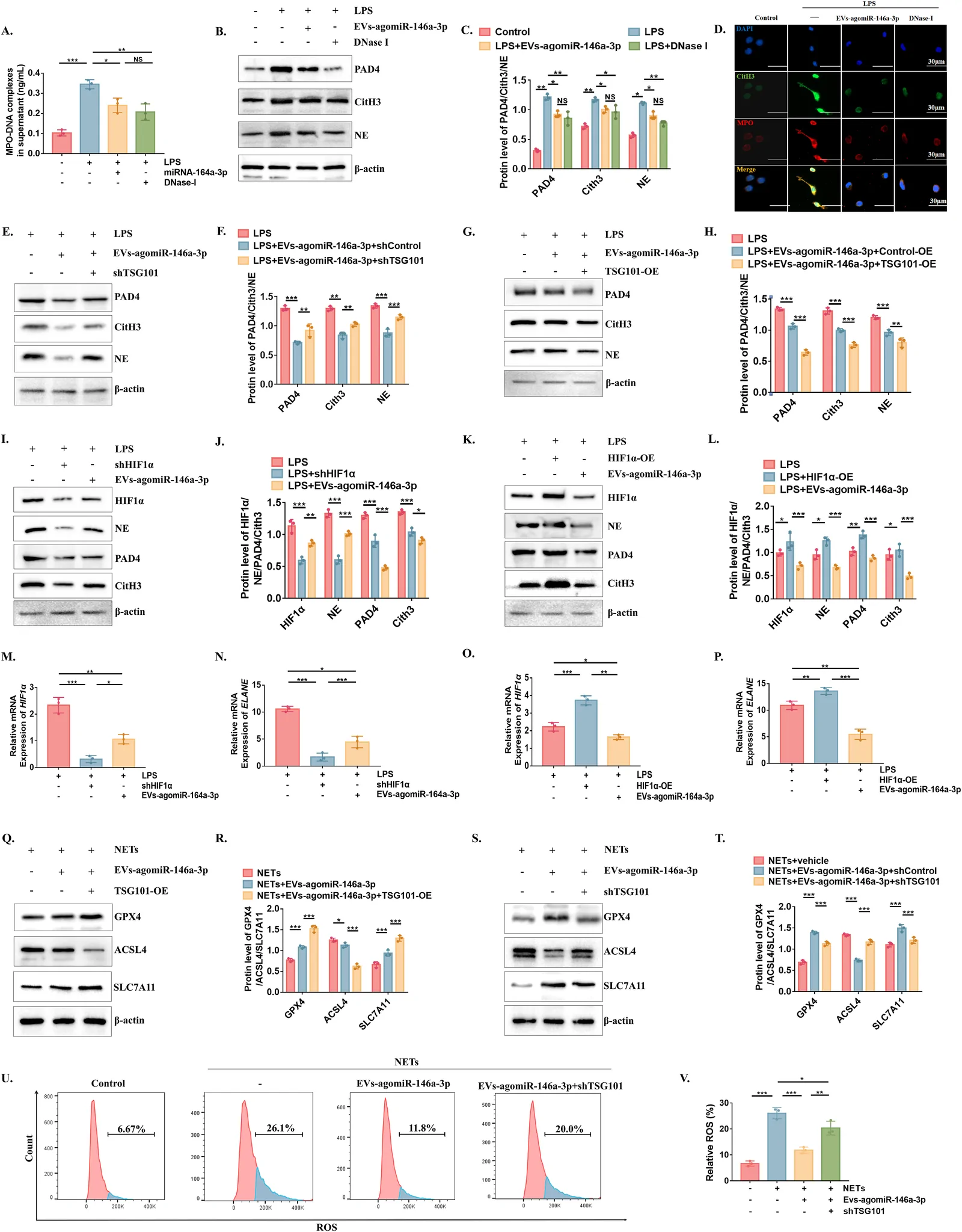
**Exosomal miR-146a-3p from BEAS-2B cells inhibits NET formation and alleviates ferroptosis by targeting HIF-1α**. **(A)** MPO-DNA complexes in supernatant. **(B-C)** Regulatory effects of EVs-agomiR-146a-3p and DNase I on the expression of LPS-induced NETs-related proteins. **(D)** The expression of MPO (red) and CitH3 (green) in dHL-60 cells was detected by immunofluorescence. Nuclei were counterstained with DAPI (blue). Scal bal = 30 μm. **(E-F)** Regulatory effects of EVs-agomiR-146a-3p and shTSG101 on the expression of LPS-induced NETs-related proteins. **(G-H)** Regulatory effects of EVs-agomiR-146a-3p and TSG101-OE on the expression of LPS-induced NETs-related proteins. **(I-J)** Regulatory effects of sh*HIF1A* and EVs-agomiR-146a-3p on LPS-induced protein expression. **(K-L)** Regulatory effects of HIF-1α overexpression and EVs-agomiR-146a-3p on LPS-induced protein expression. **(M-P)** Regulatory effects of HIF-1α knockdown and overexpression on *ELANE* and *HIF1A* mRNA expression levels. **(Q-R)** Regulatory effects of TSG101 overexpression and EVs-agomiR-146a-3p on NETs-induced ferroptosis-related protein expression. **(S-T)** Regulatory effects of shTSG101 and EVs-agomiR-146a-3p on NETs-induced ferroptosis-related protein expression. **(U-V)** Effects of shTSG101 and EVs-agomiR-146a-3p on NETs-induced ROS production. Data are presented as mean ± SD (n = 3). *P < 0.05, **P < 0.01, ***P < 0.001.

Building on our previous finding that HIF-1α drives NETs formation by targeting the *ELANE* promoter, we further examined whether exosomal miR-146a-3p interacts with this regulatory axis. Upregulation of EVs-agomiR-146a-3p significantly counteracted the HIF-1α-induced promotion of NET formation (Fig. 8I-L) and attenuated HIF-1α-mediated NE transcription (Fig. 8M-P). This suggests that miR-146a-3p may negatively regulate NET formation by interfering with HIF-1α/NE signaling at least partly.

Given the damaging effect of NETs on target cells in the inflammatory microenvironment, we further explored the impact of NETs on ferroptosis in BEAS-2B cells using a neutrophil-conditioned medium co-culture system. The results showed that the addition of NETs to the culture system led to significant abnormalities in the expression levels of ferroptosis-related proteins such as GPX4, ACSL4, and SLC7A11 in BEAS-2B cells. However, treatment with EVs-agomiR-146a-3p effectively alleviated the NETs-induced ferroptosis phenotype in BEAS-2B cells (Fig. 8Q–T). Flow cytometry analysis further confirmed that miR-146a-3p significantly reduced NETs-induced ROS release in BEAS-2B cells (Fig. 8U). Biochemical assays demonstrated that miR-146a-3p treatment significantly increased intracellular levels of reduced glutathione (T-GSH, GSH) and the GSH/GSSG ratio (Fig. S3C–F), while inhibiting the GSSG and lipid peroxidation products (4-HNE, MDA) (Fig. S3G and H), inhibiting the imbalance of lipid peroxidation.

These findings demonstrate that exosomes secreted by BEAS-2B cells deliver miR-146a-3p to neutrophils, where it blocks NETs formation via targeted inhibition of HIF-1α. The resulting reduction in NETs subsequently alleviates airway epithelial cell ferroptosis, thereby conferring a protective effect on BEAS-2B cells.

### 3.9 Exosomal miR-146a-3p from BEAS-2B Cells Relieves NETs-Exacerbated Airway Inflammation in HDM-induced asthmatic mice

To evaluate the targeting efficacy and therapeutic potential of exosomal miR-146a-3p from BEAS-2B cells, we administered the mimic via tail vein injection and assessed its pulmonary accumulation using in vivo imaging. DiI-labeled miR-146a-3p rapidly accumulated in lung tissues within 30 min post-injection, with fluorescence intensity progressively increasing up to 4 h and remaining stably retained for at least 48 h, indicating robust pulmonary targeting and sustained retention capacity of miR-146a-3p (Fig. 9A–B).

**Fig. 9:**
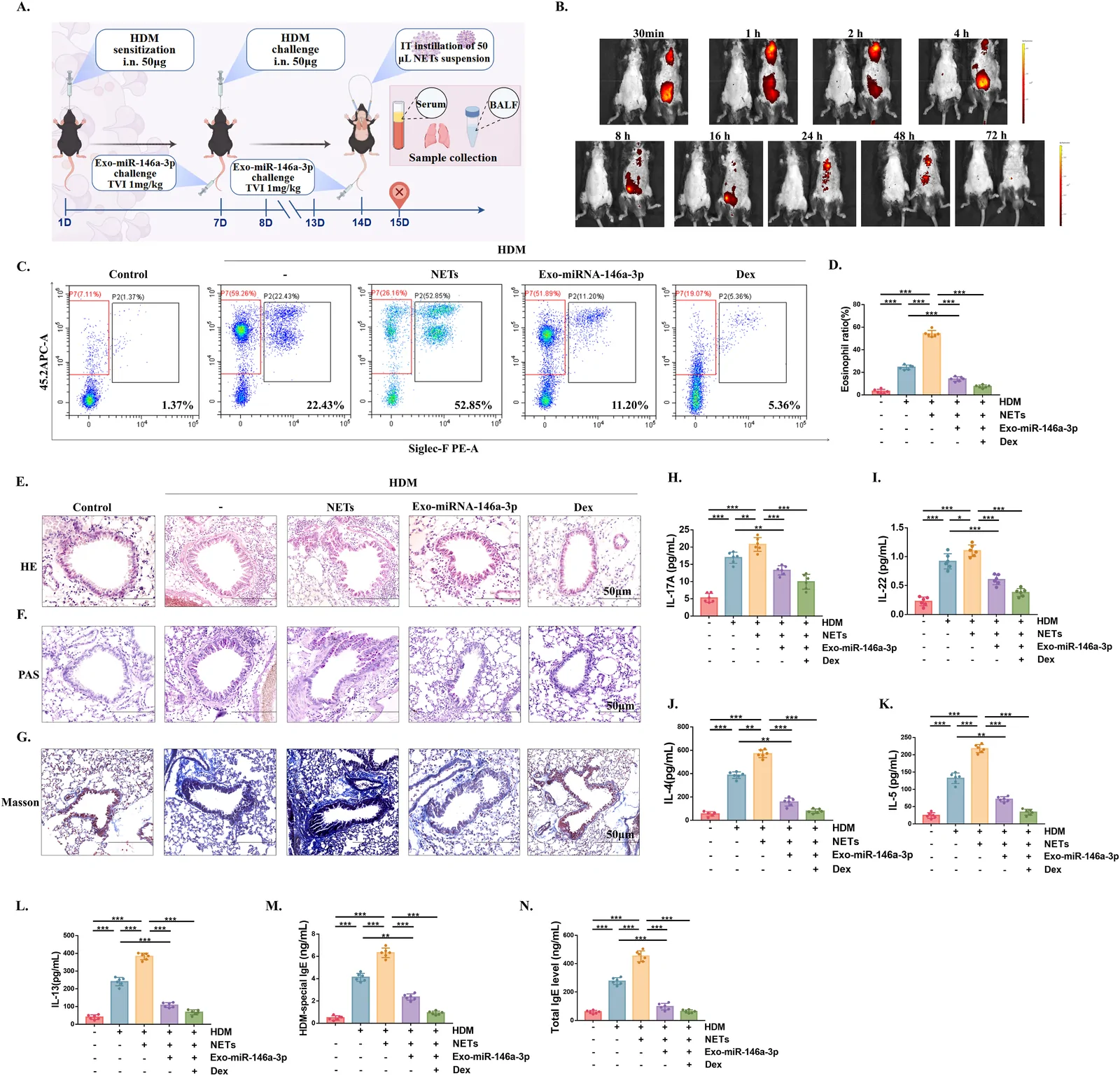
**Exosomal miR-146a-3p from BEAS-2B Cells Relieves NETs-Exacerbated Airway Inflammation in HDM-Induced Asthmatic Mice**. **(A)** In vivo imaging of exosomal miR-146a-3p targeted delivery. **(B)** In vivo fluorescence imaging of miR-146a-3p in mice. **(C-D)** Flow cytometry analysis of eosinophils in the BALF. **(E-G)** Images of H&E-stained, periodic acid-Schiff-stained and Masson-stained lung tissue sections. Scale bar = 50 μm. **(H-L)** Measurement of inflammatory cytokine expression levels. **(M-N)** Total serum IgE and HDM-specific IgE levels detected. Data are presented as mean ± SD (n = 6). *P < 0.05, **P < 0.01, ***P < 0.001.

We further explored the therapeutic potential of Exo-miR-146a-3p in restraining inflammatory cascades in vivo. Flow cytometric enumeration of eosinophils in BALF showed that NETs enhanced eosinophilic recruitment into the airway compartment, whereas Exo-miR-146a-3p intervention attenuated this NETs-fueled eosinophilic infiltration (Fig. 9C and D). Consistent with these findings, histopathological assessment revealed that exosome-encapsulated miR-146a-3p alleviated NETs-induced pulmonary inflammatory cell influx, concomitantly reducing mucus overproduction, goblet cell metaplasia, and interstitial collagen deposition in the lung tissues of asthmatic mice (Fig. 9E–G). Cytokine profiling further revealed the broad immunomodulatory activity of Exo-miR-146a-3p. Exo-miR-146a-3p not only suppressed NETs-driven induction of the Th17-signature cytokines IL-17A and IL-22 (Fig. 9H and I) but diminished the Th2-skewed inflammatory mediators IL-4, IL-5, and IL-13 (Fig. 9J–L). Quantification of total and HDM-specific IgE showed robust elevation after HDM provocation, which was further amplified by NETs challenge. Notably, Exo-miR-146a-3p delivery was sufficient to limit IgE biosynthesis and reverse HDM-elicited type 2 immune activation (Fig. 9H–N).

Collectively, these findings demonstrate that BEAS-2B-derived exosomal miR-146a-3p modulates Th17-type inflammatory responses, suppresses IgE production, and reduces eosinophilic infiltration, thereby effectively ameliorating NETs-aggravated allergic airway inflammation.

## 4 Discussion

Asthma, as a heterogeneous chronic airway inflammatory disorder, imposes a substantial burden on global public health, with its severe clinical manifestations and frequent exacerbations posing major challenges to current therapeutic strategies [43]. The complexity of its pathogenesis, particularly the intricate crosstalk between immune cells, epithelial cells, and molecular signaling pathways, remains a major obstacle to the development of effective therapies. This study elucidates a novel regulatory axis for the pathogenesis of asthma, integrating the neutrophil inflammatory response, airway epithelial damage, and programmed cell death. We propose that NETs, released by activated neutrophils, not only amplify airway inflammation but also induce ferroptosis in BEAS-2B by activating the cGAS-STING pathway. Furthermore, we identified that the airway epithelial-derived exosomal miRNA, miR-146a-3p, can negatively regulate this process by targeting HIF-1α, thereby inhibiting NETs formation and attenuating downstream ferroptotic damage. These findings provide a multi-layered mechanistic framework linking innate immune activation, transcriptional regulation, and metabolic cell death in asthma pathology.

The non-Th2, neutrophil-dominated inflammatory subtype associated with acute exacerbation remains poorly understood and lacks targeted regulatory mechanisms. In this study, systematic bioinformatics enrichment analysis overturned the conventional Th2-dominant paradigm, revealing that core asthma pathogenic genes are prominently enriched in the Th17 cell differentiation and IL-17 signaling pathways, which are well-recognized drivers of neutrophil recruitment and persistent airway inflammation [44]. Consistent with bulk RNA-seq findings, our single-cell transcriptomic profiling of clinical asthma samples provided in situ cellular evidence for this unique inflammatory and cell death regulatory landscape. Through rigorous cell clustering and immune cell annotation based on canonical marker genes, we successfully identified 14 distinct cell subpopulations in asthmatic lung tissues and precisely defined neutrophil clusters using specific signature genes, eliminating the interference of heterogeneous immune cells. The single-cell UMAP visualization distinctly captured the dynamic HIF-1α expression patterns in neutrophils during asthma progression: neutrophils before asthma onset maintained low basal HIF-1α expression, whereas during asthma development, robust HIF-1α upregulation and a sharp increase in the HIF-1α-high neutrophil subpopulation were observed. This single-cell evidence directly confirms that HIF-1α-mediated transcriptional reprogramming is a specific cellular event activated in neutrophils as the disease progresses, rather than a non-specific systemic inflammatory response, which complements and validates our bioinformatics predictions at a single-cell resolution.

Notably, previous studies on HIF-1α in asthma mainly focused on its systemic hypoxia adaptation function, lacking cell-specific and stage-specific expression characteristics [45]. Our results found that LPS-induced inflammatory stimulation drives time-dependent nuclear translocation of HIF-1α in neutrophils, which endows HIF-1α with complete transcriptional activity to initiate downstream target gene expression. Together with our in vivo results showing elevated BALF neutrophil counts and NETs-mediated neutrophil accumulation in HDM-challenged asthmatic mice, we demonstrate that the HIF-1α transcriptional activation signal specifically activated in neutrophils during acute exacerbation is a key upstream trigger for sustained neutrophil infiltration and NETs release. Moreover, single-cell sequencing analysis of risk-stratified asthma cohorts further supported the Th17-centered inflammatory mechanism driving neutrophil dysfunction. We found that high-risk asthma patients exhibited significantly elevated T cell proportions and enhanced Th17 transcriptional signatures, which is consistent with the established regulatory role of Th17/IL-17 signaling in amplifying neutrophil-mediated airway inflammation [44]. Collectively, these multi-omics and single-cell results suggested that high-risk asthma manifests enhanced Th17 inflammatory signaling, which recruits and activates neutrophils, further inducing HIF-1α nuclear translocation and transcriptional reprogramming in neutrophils to promote NETs formation, ultimately driving airway inflammatory injury and ferroptosis.

Our initial bioinformatic analysis highlighted a strong association between asthma pathogenesis and ferroptosis, aligning with previous studies that iron-dependent lipid peroxidation contributes to airway inflammation and remodeling [46]. The central finding that NETs serve as a potent inducer of ferroptosis expands the function of NETs beyond direct cytotoxicity and immune activation. NETs have long been recognized as critical mediators of inflammation in diseases such as rheumatoid arthritis [47] and ARDS [48], but their ability to orchestrate a specific form of metabolic cell death adds a new dimension to our understanding of neutrophil-driven pathology in asthma. More importantly, NETs act as crucial upstream signaling molecules that precisely activate the cGAS-STING pathway in airway epithelial cells. It is particularly noteworthy that this activation mode is distinctly different from the classical type I interferon response pathway in that it does not rely on the TBK1/IRF3 phosphorylation-mediated signaling cascade [27], but rather drives downstream characteristic events of ferroptosis by regulating the total expression levels of core pathway proteins such as cGAS, STING, TBK1, and IRF3. These events manifest as downregulation of GPX4 and SLC7A11 expression, upregulation of ACSL4 expression, disruption of glutathione redox homeostasis, and accumulation of lipid peroxidation products. This discovery revealed that cGAS-STING pathway from its traditional role as a "central hub of antiviral defense", it now extends to a "key regulatory node mediating metabolic cell death" [49]. Simultaneously, by releasing NETs, neutrophils can directly deliver a "death instruction" to adjacent airway epithelial cells, diverting them from conventional cell death pathways and specifically initiating ferroptosis, a metabolism-dependent death program. This process tightly couples acute inflammatory signals with irreversible airway tissue injury and remodeling.

MiR-146a is a well-established inflammation-associated miRNA, often acting as a feedback inhibitor of TLR/NF-κB signaling [50]. Although miR-146a-5p has been well-documented as a potent anti-inflammatory mediator in allergic airway diseases, considerably less attention has been paid to its sibling strand miR-146a-3p, which is derived from the same pre-miR-146a precursor transcript. Previous studies have demonstrated that miR-146a-5p predominantly restrains type-2 allergic responses by targeting key adaptor molecules within the TLR-NF-κB cascade and curbing ILC2-driven inflammatory signalling in asthmatic airways [16]. In contrast, our discovery that it directly targets HIF-1α for downregulation reveals a distinct mechanism. HIF-1α is a master regulator of cellular adaptation to hypoxia and inflammation, and its involvement in promoting NETosis is supported by our and others' work [51]. By suppressing HIF-1α, miR-146a-3p disrupts its transcriptional activation of *ELANE*, a critical effector in NETs formation. This creates a paracrine loop wherein epithelial cells, sensing or responding to an inflammatory milieu, release exosomes carrying miR-146a-3p to dampen neutrophilic hyperactivation. The dependence of this process on the exosomal protein TSG101 underscores the specificity of this vesicle-mediated delivery. These two mature miRNAs exhibit divergent target repertoires and cell-type-specific functional outputs: miR-146a-5p mainly acts on innate immune lymphocytes to constrain Th2-type inflammation, whereas exosome-delivered miR-146a-3p modulates neutrophil pathogenic programmes to interrupt the NETs-cGAS-STING-ferroptosis axis. Such functional divergence highlights that distinct arms from the same miRNA precursor can participate in separate pathological modules of asthma.

It should be emphasized that the HDM-induced mouse model, though widely used [52], may not fully recapitulate the heterogeneity and complexity of human asthma. Notably, interspecies differences in neutrophil biology, HIF-1α regulatory networks and ferroptosis responsiveness exist between mice and humans. Validation in clinical samples such as BALF and peripheral blood is therefore warranted. Nevertheless, the relative inaccessibility of these specimens may present practical challenges for clinical investigation. A further consideration is that the therapeutic potential of exosomal miR-146a-3p still requires optimized delivery and stability, as well as evaluation of its safety and efficacy in preclinical and clinical settings.

## 5 Conclusion

In conclusion, our study delineates a novel regulatory network in asthma, wherein miR-146a-3p, delivered via BEAS-2B-derived exosomes, targets HIF-1α to inhibit NETs formation. NETs, in turn, activate the cGAS-STING pathway to induce ferroptosis in airway epithelial cells, exacerbating airway inflammation and tissue damage. It provides a comprehensive mechanistic framework for understanding the pathogenesis of asthma and identifies potential therapeutic targets. Exosomal miR-146a-3p, in particular, holds great promise as a targeted therapy for asthma by modulating multiple key nodes of this pathogenic pathway. These findings advance our understanding of asthma pathogenesis and lay the groundwork for the development of more effective, personalized therapies for this challenging disease.

## CRediT authorship contribution statement

**Jianing Yang:** Writing – review & editing, Writing – original draft, Software, Project administration, Methodology, Investigation, Data curation. **Yunong Li:** Writing – review & editing, Software, Methodology. **Zhien Li:** Writing – review & editing, Methodology. **Mingyue Liu:** Software, Methodology. **Yedong Xiang:** Software, Methodology. **Feifei Xu:** Methodology. **Yulian Zhang:** Methodology, Formal analysis. **Guanhao Li:** Formal analysis. **Jingmei Chai:** Visualization, Funding acquisition. **Guanghai Yan:** Supervision, Funding acquisition, Conceptualization. **Chongyang Wang:** Writing – review & editing, Software, Project administration, Funding acquisition, Data curation, Conceptualization.

## Ethics declaration

Written informed consent to take part in the study and to publish the article has been obtained from all participants or their legal representatives. The privacy rights of participants have been observed.

This study was performed in compliance with relevant laws, regulatory frameworks and guidelines where the research took place. This study was conducted in accordance with This study used anonymized public human omics datasets from the GEO database. The original studies obtained ethical approval and informed consent for data deposition. The analysis complied with the Declaration of Helsinki. Ethics committee approval was not required under relevant laws and institutional guidelines. The in vitro cell experiments and bioinformatic analyses did not involve human participants or human biological specimens.

This study was conducted in accordance with the ARRIVE (Animal Research: Reporting of In Vivo Experiments) guidelines. This study was approved by the Animal Care and Utilization Committee of Yanbian University. (Approval No. YDSY20260814001)

## Availability of data and materials

All data generated or analyzed during this study are included in this article and its Additional file.

## Declaration of competing interest

The authors declare that they have no known competing financial interests or personal relationships that could have appeared to influence the work reported in this paper.

## Figures and Tables

**Supplementary 1:**
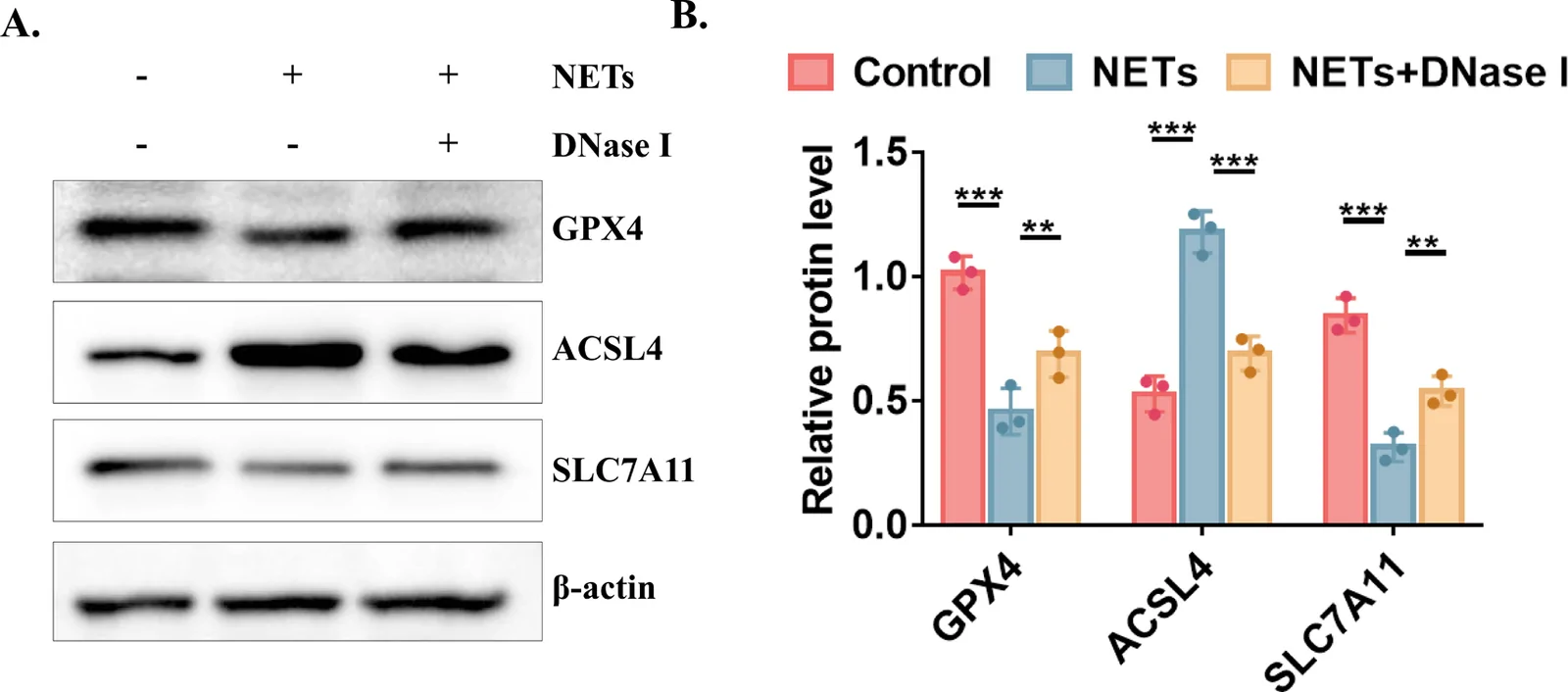
**(A-B)** Western blotting analysis of ferroptosis-related protein levels in BEAS-2B cells was performed. Data are presented as mean ± SD (n = 3). *P < 0.05, **P < 0.01, ***P < 0.001.

**Supplementary 2:**
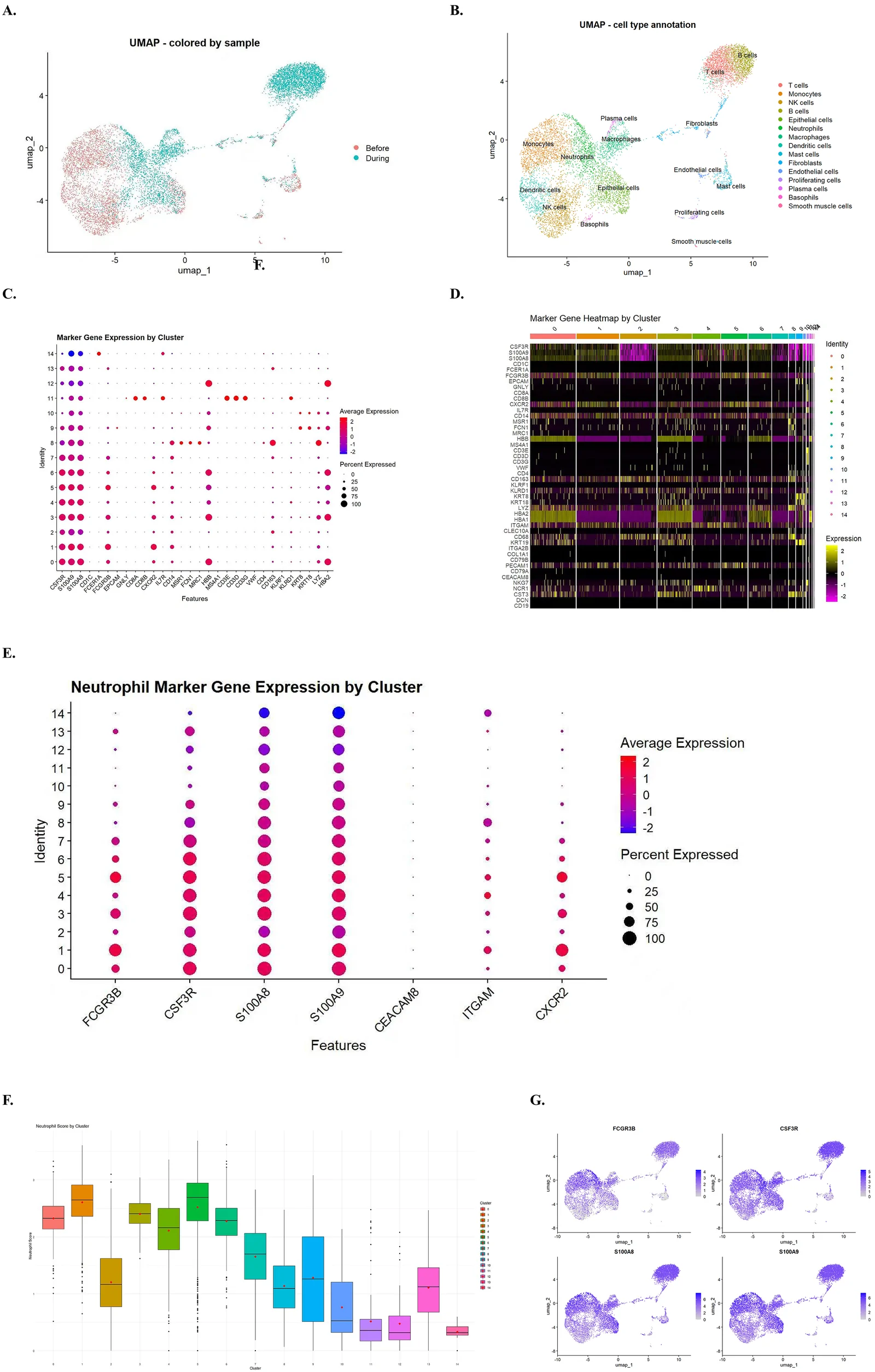
**(A)** UMAP visualization of single-cell transcriptomes from paired lung tissue samples of asthmatic patients collected before (red) and during (cyan) an asthma episode. Each dot represents an individual cell (n = 1 patient). **(B)** UMAP visualization of major cell types in human lung samples. Cells are colored by 15 annotated clusters. **(C)** Dot plot showing the scaled average expression (color intensity; red = high, blue = low) and the percentage of expressing cells (dot size) of canonical marker genes across the identified cell clusters. **(D)** Heatmap displaying the scaled expression (yellow = high, purple = low) of marker genes across all identified cell clusters. Each column represents an individual cell, grouped by cluster. **(E)** Dot plot showing the expression of specific neutrophil marker genes (FCGR3B, CSF3R, S100A8, S100A9, CEACAM8, ITGAM, CXCR2) across the clusters. **(F)** Boxplot showing the neutrophil signature score across all identified cell clusters. Boxes denote the interquartile range (IQR) with the median line, red dots indicate the mean score, and black dots represent outliers. **(G)** UMAP feature plots showing the normalized expression of neutrophil marker genes (FCGR3B, CSF3R, S100A8, and S100A9). Color intensity indicates expression levels (dark blue = high, gray/purple = low). Abbreviations: UMAP, Uniform Manifold Approximation and Projection; IQR, interquartile range.

**Supplementary 3:**
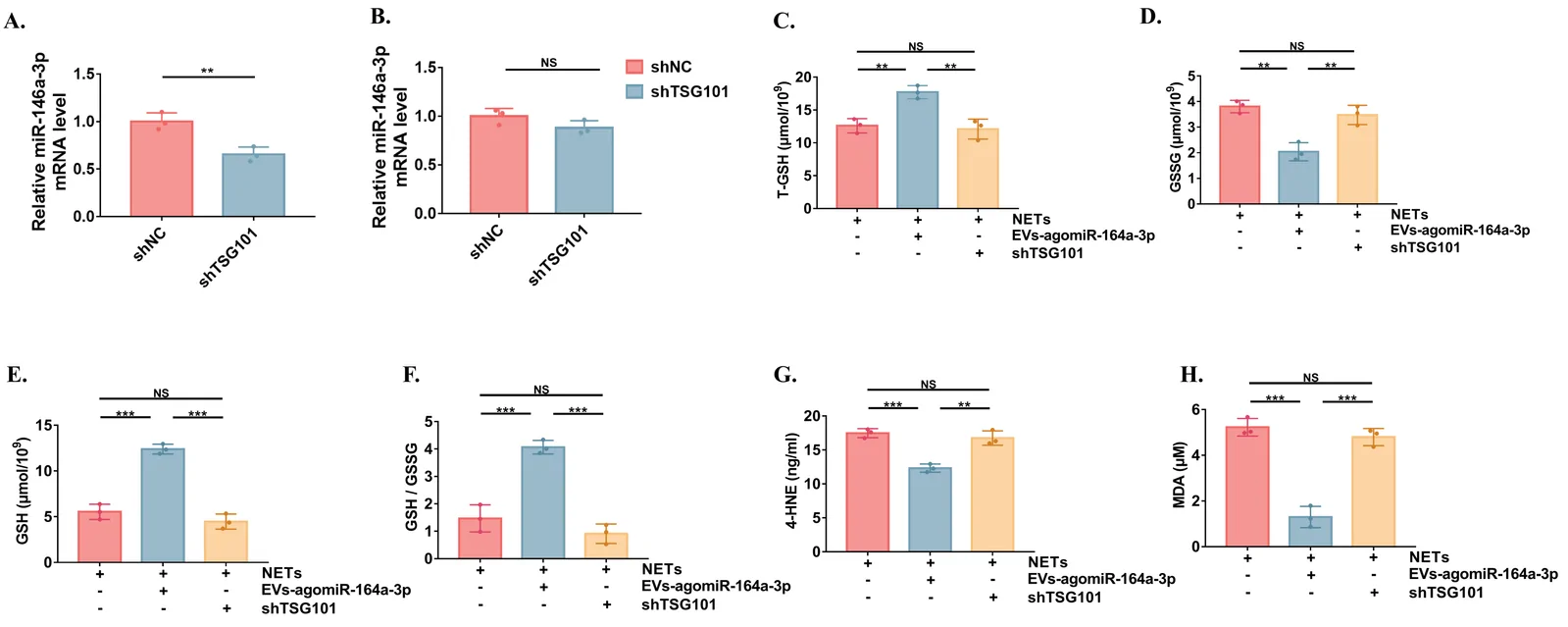
**(A)** qRT-PCR was used to detect the relative expression of miR-146a-3p in exosomes harvested from shNC and shTSG101 groups. **(B)** qRT-PCR was applied to detect the relative miR-146a-3p expression in cells of shNC and shTSG101 groups. **(C-F)** The levels of T-GSH, GSSG, GSH, and GSH/GSSG were measured by T-GSH/GSSG colorimetric assay kit. **(G)** The level of 4-HNE were measured by 4-HNE ELISA kit. **(H)** The level of MDA were measured by MDA assay kit. Data are presented as mean ± SD (n = 6). *P < 0.05, **P < 0.01, ***P < 0.001.

## Data Availability

Data will be made available on request.
